# Targeting Amyloid Aggregation: An Overview of Strategies and Mechanisms

**DOI:** 10.3390/ijms19092677

**Published:** 2018-09-09

**Authors:** Sofia Giorgetti, Claudio Greco, Paolo Tortora, Francesco Antonio Aprile

**Affiliations:** 1 Department of Molecular Medicine, Institute of Biochemistry, University of Pavia, Via Taramelli 3b, 27100 Pavia, Italy; sofia.giorgetti@unipv.it; 2 Department of Earth and Environmental Sciences, University of Milano-Bicocca, Piazza della Scienza 1, 20126 Milano, Italy; claudio.greco@unimib.it; 3 Department of Biotechnologies and Biosciences, University of Milano-Bicocca, Piazza della Scienza 2, 20126 Milano, Italy; 4 Milan Center for Neuroscience (Neuro-MI), 20126 Milano, Italy; 5 Centre for Misfolding Diseases, Department of Chemistry, University of Cambridge, Cambridge CB2 1EW, UK

**Keywords:** amyloid diseases, biocomputing, drug design, natural antiamyloids

## Abstract

Amyloids result from the aggregation of a set of diverse proteins, due to either specific mutations or promoting intra- or extra-cellular conditions. Structurally, they are rich in intermolecular β-sheets and are the causative agents of several diseases, both neurodegenerative and systemic. It is believed that the most toxic species are small aggregates, referred to as oligomers, rather than the final fibrillar assemblies. Their mechanisms of toxicity are mostly mediated by aberrant interactions with the cell membranes, with resulting derangement of membrane-related functions. Much effort is being exerted in the search for natural antiamyloid agents, and/or in the development of synthetic molecules. Actually, it is well documented that the prevention of amyloid aggregation results in several cytoprotective effects. Here, we portray the state of the art in the field. Several natural compounds are effective antiamyloid agents, notably tetracyclines and polyphenols. They are generally non-specific, as documented by their partially overlapping mechanisms and the capability to interfere with the aggregation of several unrelated proteins. Among rationally designed molecules, we mention the prominent examples of β-breakers peptides, whole antibodies and fragments thereof, and the special case of drugs with contrasting transthyretin aggregation. In this framework, we stress the pivotal role of the computational approaches. When combined with biophysical methods, in several cases they have helped clarify in detail the protein/drug modes of interaction, which makes it plausible that more effective drugs will be developed in the future.

## 1. Introduction

Proteins generally require specific three-dimensional conformations in order to be soluble and function correctly in the body. Under stress conditions, normally soluble proteins can undergo structural changes and self-assembly that lead to their aggregation into insoluble deposits, referred as amyloids [1,2].

Amyloids from different proteins share several structural properties: they all have a fibrillar morphology and cross-β structure, whereby intermolecular main-chain hydrogen bonding acts as one major stabilising interaction [1,3]. Frequently, they also have repetitive hydrophobic or polar interactions along the fibril axis [3]. They are highly rigid [4], resistant to thermal [5] and chemical denaturation and degradation [6].

Recent technological advances in structural biology, which include solid-state nuclear magnetic resonance (SSNMR) [7], and cryo electron microscopy (cryo-EM) [8], allowed scientists to determine the structure of amyloids at the molecular level directly from patient tissues. These studies have shown that amyloid aggregates formed in vivo consist of differently modified variants of the amyloidogenic protein and are associated and often co-aggregated with components of the protein homeostasis system, such as molecular chaperones [9].

The presence of amyloids is usually related to pathological conditions generally called amyloidoses [1]. Amyloidoses can be either localized or systemic, according to whether or not the amyloidogenic protein aggregates are in the site of synthesis, respectively, and they can have characteristic molecular and clinical hallmarks, depending on the site of deposition [10]. Neurodegenerative diseases including Alzheimer’s (AD) and Parkinson’s diseases (PD) and corea of Hungtington (CH) represent a highly prevalent class of fatal localized amyloidoses in which amyloid deposits form in the nervous system where they induce the death of specific neuronal cell types [3]. In systemic amyloidoses, such as immunoglobulin light chain (AL), transthyretin (TTR), and dialysis-related amyloidoses (DRA), several organs are affected as the amyloidogenic protein is distributed in different sites of the body while it travels from the site of synthesis [10].

The formation of amyloids depends on extremely complicated aggregation processes, in which various aggregation intermediates form through a combination of simultaneous microscopic events [11], namely: (1) *primary nucleation*, in which initial small soluble aggregates form from monomers interacting in solution; (2) elongation, in which existing fibrils increase in length by monomer addition; (3) secondary nucleation processes, in which the surface of existing aggregates catalyses the formation of new small soluble aggregates and (4) fragmentation, in which existing fibrils break apart, increasing the total number of fibrils [12]. The contributions of each of these microscopic events to the lag and growth phases are highly protein specific.

Small soluble aggregates formed during the amyloid aggregation are generally called oligomers. These protein species are extremely heterogeneous and can rapidly interconvert into protofibrils. They are extremely toxic and believed to play a major role in cell and tissue toxicity, particularly in neurodegenerative diseases [13,14,15,16]. The mechanisms of toxicity of amyloid oligomers are still under debate. In vivo and in vitro studies have demonstrated that high levels of amyloid oligomers are able to over-stimulate glutamatergic synaptic transmission and cause synapse loss [17,18,19,20,21,22]. It has also been reported that oligomers are able to interact with the cell membrane [16,23] and are associated with oxidative stress [14,24], altered calcium homeostasis (which is the most sensitive to alterations of membrane permeability, due to its huge concentration gradient across the plasma membrane, i.e., about four orders of magnitude) [25], mithocondrial dysfunction [26], and inflammation [27].

In the case of systemic amyloidoses, the mechanisms of toxicity are probably different, and both insoluble fibrils and soluble oligomers are important for cytotoxicity [28]. For example, it has been reported that a further pathogenic effect besides that played by oligomers results from the massive extracellular accumulation of amyloid fibrils, which cause mechanical stress, and ultimately organ function impairment and failure [10].

In neurodegenerative diseases, the amyloidogenic proteins are often intrinsically disordered, which means that no misfolding event needs to occur to initiate their aggregation. Instead, the transition to amyloid is generally directly triggered by mutations, post-translational modifications (such as proteolytic cleavages or chemical modification of the protein side chains or backbone), and interactions with the environment, such as membranes and lipids [29,30].

Most of the theoretical body on the mechanism of amyloidogenesis and amyloid toxicity for systemic amyloidoses derives from experimental studies carried out on three types of proteins: LC, TTR and β2-microglobulin (β2-m) [31]. A generic step for the amyloid transition of these globular proteins and the acquisition of cytotoxic properties is the partial unfolding of the native state. Also in this case, mutations and selective proteolytic cleavages are certainly two major determinants in facilitating the amyloid transition. Studies on β2-m were particularly informative because the clinical counterpart of the experimental and theoretical side is relatively simple [32], at least simpler than for TTR and immunoglobulin. There are only two types of amyloidoses caused by β2-m, one acquired and one genetically transmitted, and both extensively characterized.

Dialysis-related amyloidosis is the acquired form of the disease and is caused by a substantial increase in monomeric β2-m plasma concentration resulting from haemodialysis. As a consequence, the protein acquires a strong propensity to misfold and aggregate. Normally, the intrinsic propensity to misfold is inhibited by the stabilizing interaction with the heavy chain within the MHC I and this observation offers the natural demonstration that amyloidogenesis can be prevented by stabilization through protein/protein or protein/ligand interactions. There is, so far, only one reported mutation associated to β2-m amyloidosis occurring in the absence of haemodialysis and low concentration of circulating β2-m. The study of the mutation Asp76Asn has disclosed a new scenario because it led to the discovery that the partial unfolding and amyloid transition can be obtained by simply playing through the biomechanical forces generated by the turbulent fluid flow of a physiologic fluid at the interface with hydrophobic patches [33]. Such a discovery unleashed a flurry of research aimed at designing new biocompatible models of in vitro fibrillogenesis for this and other proteins such as TTR [34], which are especially suitable for understanding the in vivo mechanism of amyloidogenesis and offering a reliable tool for drug discovery.

**Figure 1:**
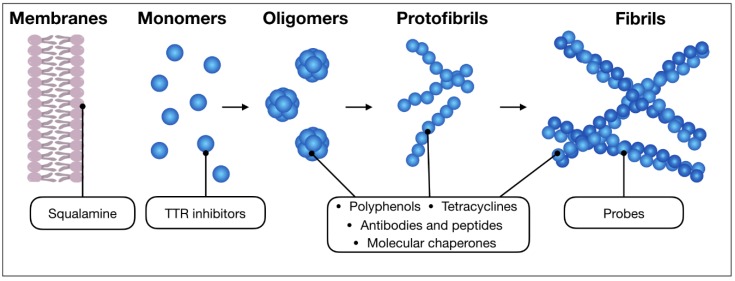
Schematic representation showing the intermediates of a generic amyloid aggregation pathway (monomers, oligomers, protofibrils and fibrils). The scheme includes a membrane as well, which in some cases can play a role in the process, such as for α-syn. In the figure, the main classes of anti-aggregation molecules discussed in this review are connected to the aggregated species to which they have been reported to preferentially bind.

**Figure 2:**
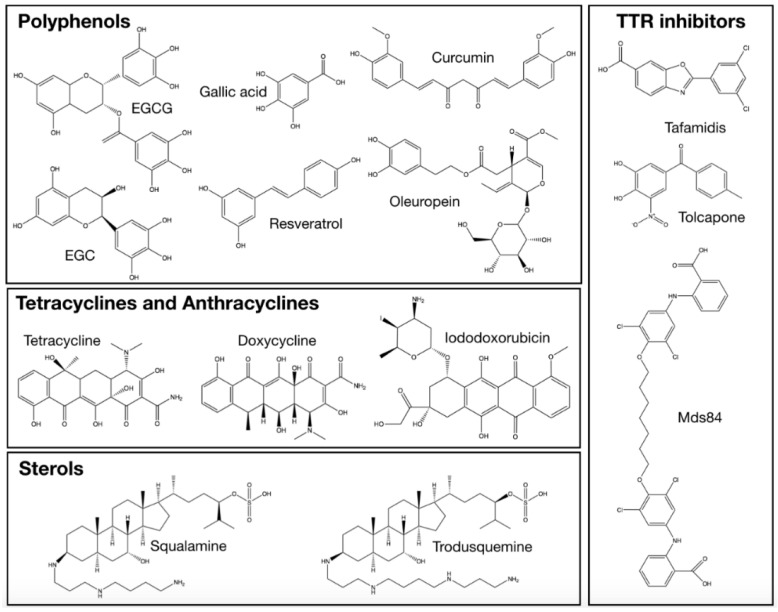
Chemical structures of the antiamyloid compounds discussed in the present review.

The present review will focus on and discuss effects and therapeutic efficacy of drugs and nutraceuticals currently in use or under investigation, which are endowed with a well-documented capability of inhibiting the appearance of toxic protein aggregates. In doing so, we mainly aim at highlighting the methodological aspects related to the mechanisms of action of such compounds and to the development of new ones, rather than providing a comprehensive survey of this topic, provided this will be ever possible. In particular, their mode of interaction with the proteins committed to amyloidogenesis will be analysed. It should be stressed, however, that many such compounds also act by mitigating some of the aforementioned toxic effects at the cellular level. In any case, evidence of beneficial effects precisely fulfilled at the level of protein aggregation will be presented and discussed, when available. In Figure 1, the general features of the amyloidogenic pathway are shown, also highlighting the step(s) where these compounds interfere with the process, at least in most cases. In Table 1, a wide compilation thereof is provided, whereas the formulas of those compounds, which will be discussed in the present review are presented in Figure 2.

Finally, in the last chapter we will highlight how in vitro and in silico approaches have contributed to the present knowledge and how they have complemented each other.

## 2. The Main Classes of Anti-Amyloid Compounds

### 2.1. Probes and Diagnostic Molecules

Since the origins of the amyloid field, several compounds have been developed for research purposes in order to characterise the mechanisms of formation and structure of amyloids. In particular, many structural analyses are currently based on the use of molecular probes that change their spectroscopic properties upon binding to the amyloid fibrils. This is the case of thioflavin-T (ThT), a fluorescent molecule that is now routinely used for monitoring the time evolution of the amyloid aggregates in vitro [80] or the compounds 1-anilinonaphthalene-8-sulfonic acid (ANS) and 4,4′-dianilino-1,1′-binaphthyl-5,5′-disulfonic acid (bisANS), which are employed in a similar way to ThT. However, as they recognise solvent-exposed hydrophobic patches of proteins, they are also used for characterising early-stage oligomerization and initial structural rearrangements of amyloidogenic proteins [81]

Furthermore, an increasing research effort has now focused on improving the affinity of these molecules in order to develop high sensitivity methods for the study of amyloids. For example, it has been show that ThT dimers can have a 70-fold higher affinity for amyloid fibrils than the original molecule while maintaining its fluorescent properties and binding selectivity [82]. The development of molecules of this type will facilitate in vitro studies of amyloids at nanomolar concentration, which represents a more relevant condition for characterizing their mechanisms of toxicity.

There are several other molecules, such as Thioflavin S or [50-(*p*-Hydroxyphenyl)-2,20-bithienyl-5-yl]-methylidene}-propanedinitrile (NIAD-4) [83], which have been successfully employed for diagnostic and research applications ex vivo or on tissues thanks to their selective binding to amyloid aggregates. Some of these molecules (Table 1) have also been proved to affect amyloid aggregation to different extents and with different mechanisms.

Congo red (CR) is probably the most famous case. This molecule is used for determining the amyloid nature of protein aggregates from biological samples. In particular, CR shows green birefringence under polarized light in the presence of amyloid aggregates. Recent studies have shown that CR is able to inhibit the aggregation of a series of proteins, including amyloid beta (Aβ), casein, the prion protein (PrP), α-syn (α-synuclein) [52,53]. In particular, CR is able to accelerate these aggregation processes, thus reducing the life-time of toxic oligomeric species [53].

Another relevant compound in this category is crystal violet, which can be used for the detection of amyloid aggregates in histologic preparations for light microscopy. This molecule has been reported to an effective inhibitor of tau aggregation [50]. Structurally similar molecules such as acid fuchsin and fast green FCF have been reported to have the same effect [55].

It is also worth mentioning methylene blue, a phenothiazine used as a treatment for haemoglobin conditions and as stain for cells and tissues in endoscopic procedures. While not specifically designed to be a fibril probe, this molecule has anti-amyloid effects. In particular, it has been shown that methylene blue can inhibit the oligomerization of amyloidogenic proteins with different mechanisms. More specifically, in the case of PrP, methylene blue inhibits the formation of oligomers by affecting its fibrillization [78]. In the case of Aβ, methylene blue increases fibrillization of the peptide, depleting monomers available for oligomerization [79].

### 2.2. Anthracyclines and Tetracyclines

In 1995, staring at the clinical observation that the anthracycline 4′-iodo-4′-deoxy-doxorubicin (IDOX) was able to induce amyloid resorption in patients with AL amyloidosis, Merlini et al. showed the capability of this drug to interact with several types of amyloid fibrils and to inhibit the amyloid conversion of native proteins [46,47]. However, due to its intrinsic cardiotoxicity, the clinical exploitation of the drug was discontinued.

The search for structural analogues of IDOX resulted in the identification of tetracyclines as good candidates for mimicking the IDOX activity despite the lack of significant cardiotoxicity. A confirmation of the hypothesized anti-amyloid efficacy of tetracyclines came from experiments on inhibition of PrP infectivity in animal models [84].

The generic effect of tetracyclines in interfering with amyloid formation inspired further investigation on the mechanism of interaction with amyloid structure and consequent blocking of amyloid growth.

Through a molecular mechanic approach, Cosentino et al. highlighted the crucial role of the hydrophobic core given by aromatic rings in the generic interaction with amyloid [85]. This study provided insight into how different polar substituents could determine the specificity of the interaction between various analogues of tetracyclines with different types of fibrils. The fact that the affinity for tetracyclines differs from fibril to fibril is most likely based on the structural heterogeneity and polymorphisms of fibrils now clearly emerging from their structure solved at the atomic level by solid state NMR and cryo-EM [86]. Thus, the overall picture of the drug’s mode of action that emerges from available data is multifaceted. Apparently, tetracyclines not only bind mature fibrils, but can also interact with soluble precursors of insoluble amyloid fibrils: monomers and oligomers. In the case of the Aβ peptide, tetracyclines bind oligomers, but not the monomer [38]; in the case of the globular protein β2-m the binding not only involves oligomers but also the monomer through a binding site highly influenced by the physical-chemical properties of the environment [39]; furthermore, in the case of ataxin-3 (ATX3), tetracycline only binds oligomers via functional groups, mostly hydrophobic, located on one edge of the molecule, probably shielding to some extent the aggregate from the medium [87,88]. It is worth noting that also fibrils, upon binding to tetracyclines, deeply rearrange their structure resulting in the formation of disordered insoluble material lacking the typical features of amyloid fibrils [40].

Regardless of the molecular target and mechanism of binding, the capacity of tetracyclines to inhibit the intrinsic toxicity of these soluble conformers is apparently due to the drugs’ capability to structurally rearrange the toxic oligomers [41], thus converting them into inactive molecules.

The best investigated type of tetracyclines is doxycycline (DOX), not only because is one of the most effective conformers on several type of fibrils in vitro, but also because it has been used in vivo for many years as a wide-spectrum antibiotic with no appreciable adverse effects. Its use in amyloidosis just represents the repurposing of an old drug on a new target.

Based on the anti-amyloid properties demonstrated in vitro, the clinical efficacy of DOX is now under investigation in at least three types of systemic amyloidoses (http://clinicaltrials.gov). In TTR-related amyloidosis a phase-3 clinical trial is in the stage of patients recruitment. In this study, DOX is used in combination with Tauroursodeoxycholic acid and the trial is designed on the basis of the results of a previous phase-2 study, showing the efficacy of this treatment in stabilizing the disease [42].

DOX was used in an exploratory off-label study on three patients affected by a severe form of dialysis-related amyloidosis (DRA) and although the amyloid mass was not apparently reduced, the patients experienced a very significant reduction of the ostheoarticular pain, as well as a remarkable improvement of the active and passive movements [43]. Although the mechanism in vivo is not clarified, the benefits of this treatment were recently confirmed by Piccoli et al., who recommend the DOX treatment as antalgic therapy for this kind of patients [44]. Although clinical trials for the validation of the treatment of this amyloidosis are not currently ongoing, DOX has received the designation of orphan drug by the European Medicines Agency for the treatment of DRA and hopefully a trial will be designed soon because there is no treatment for this very debilitating disease.

Several clinical trials are now active or in the pipeline in AL amyloidosis caused by the fibrillar deposition of immunoglobulin light chains. In these trials, the purpose is to evaluate potential benefits on the disease outcome by the addition of DOX to standard chemotherapy used in these patients. These studies were strongly encouraged by the data reported by Wechalekar et al. [45], showing that addition of DOX to standard chemotherapy significantly reduced the mortality in patients in advanced state of the disease.

### 2.3. Sterols

Sterols are a class of steroids, which are naturally produced by several organisms, including plants and bacteria. In particular, a broad-spectrum of them has been isolated from the dogfish shark *Squalus acanthias*, initially for their antibiotic properties against both Gram-negative and Gram-positive bacteria, and fungicidal anti-protozoa activity [89]. Among these molecules, the compound squalamine has been proved to be effective against cancer [90] and, very recently, against PD. In particular, squalamine is able to inhibit the aggregation of the protein α-syn [35], whose deposition into Lewy bodies in a hallmark of PD [91]. In this regard, the mechanism of action of squalamine is an example of indirect effect of a molecule on the aggregation of an amyloidogenic protein. α-syn is known for being very soluble at normal pH, even at very high (mM) concentrations. In order to aggregate, α-syn requires the presence of hydrophobic surfaces, such as lipid membranes, where α-syn monomers are attracted to and nucleate [30]. Squalamine has been proved to inhibit α-syn aggregation by displacing monomers from the membranes [35].

Recently, a squalamine derivative, called trodusquemine, has been shown to affect the aggregation of α-syn as well [36]. In addition to displacing α-syn monomers similar to the mechanism fulfilled by squalamine, trodusquemine directly interacts with α-syn to inhibit the secondary nucleation of aggregation [36].

### 2.4. Peptides and Engineered Antibodies

One very demanding goal when designing anti-aggregation compounds is the development of highly specific molecules [92]. For this purpose, scientists have then looked at molecular biology and protein engineering as a solution in order to generate peptides and proteins for therapeutic applications.

In particular, small peptides, generally referred to as β-sheet breakers or simply β-breakers, have been reported to affect the formation and stability of amyloid aggregates [93]. β-breakers are soluble short sequence portions of amyloidogenic proteins. As protein aggregation is a self-assembly process, β-breakers interact with the same sequences within amyloidogenic proteins blocking their aggregation or promoting the disaggregation of existing fibrils [93]. They have been shown to be effective in vitro in the case of Aβ [94]. Nevertheless, they are poorly stable, in vivo, being prone to proteolytic degradation and having a relatively short half-life [95,96]. To overcome these limitations, scientists are trying several chemical modifications including *N*-methylation, the incorporation of unnatural amino acids, and cyclization [97].

Small engineered protein domains can act as potent inhibitors of amyloid aggregation as well. This is the case, for example, for some antibody mimetics, in particular some affibodies, which mimic the high affinity binding of antibodies, while being structurally distinct. Among them, β-wrapins have been reported to stabilize amyloidogenic proteins in β-hairpin conformations, thus preventing self-assembly or promoting the disaggregation of preformed oligomers [98]. These molecules have been proven to be effective in inhibiting the aggregation of Aβ, α-syn, and IAPP [98,99,100].

Antibodies and antibody fragments also have anti-aggregation properties. In particular, monoclonal antibodies probably represent at the moment the class of protein therapeutics with the most positive recent result from clinical trials. For example, the antibodies Aducanumab and BAN2401 [70,71] have successfully passed phase 2 clinical trials in the context of passive immunotherapy protocols against Alzheimer’s and Parkinson’s diseases, with Aducanumab currently giving positive results in phase 3 clinical trials where it shows dose-dependent clearance of amyloid deposits and slows down cognitive decline.

Also, antibody fragments have been proved to be effective anti-aggregation molecules. For example, camel single domain antibodies, generally referred as nanobodies, are extremely effective inhibitors of the aggregation of several amyloidogenic proteins, including lysozyme [63], α-synuclein (α-syn) [64], Aβ [65], tau [101], and β2-m [66]. They have also been proved to be effective diagnostic tools for distinguishing amyloid fibrils at different maturation stages [102]. The so-called grafted amyloid-motif antibodies (or gammabodies) represent a valuable alternative class of anti-aggregation antibodies. Gammabodies are single domain human antibodies, where the complementarity-determining regions are replaced by aggregation-prone sequences from amyloidogenic proteins [67]. They then act as β-breakers with the advantage of being more soluble thanks to the stabilizing effect provided by the presence of the single-domain antibody scaffold. In addition, recent advances have disclosed new possibilities for the rational development of anti-aggregation antibody molecules [68]. In this regard, single domain antibodies have been rationally designed to specifically inhibit the aggregation of α-syn, amylin (the causative agent of islet amyloid in type-2 diabetes, IAPP) and Aβ [68,69].

Rational design has also been applied to other classes of proteins, such as molecular chaperones. They are very well known for being naturally occurring effective inhibitors of protein aggregation [9,103,104], but also for being highly non-specific, as they interact with any solvent-exposed protein hydrophobic patch. In order to increase their specificity towards amyloidogenic proteins, scientists have designed chaperone variants carrying peptides, which selectively interact with a given protein when found in aggregated conformation [105,106].

### 2.5. Polyphenols

Polyphenols are a class of compounds whose structure is characterized by the presence of several phenol units. They include a wealth of structurally diverse molecules, although they also share in part the mechanisms of action. Besides their capability to prevent or retard amyloid aggregation, several additional effects have been assigned to them, which are beneficial for human health. Most notably, they are endowed with antioxidant and anticancer properties, the latter being mediated by inhibition of antiangiogenesis. The main types of polyphenols are discussed below.

#### 2.5.1. (−)-Epigallocatechin-gallate (EGCG) and Related Compounds

EGCG is the major catechin found in the leaves of green tea. They also contain a variety of related, structurally simpler molecules, in particular (−)-epigallocatechin (EGC) and gallic acid (GA), whose effects are qualitatively similar to those exerted by EGCG [107,108].

Current literature shows ECGC’s capability to prevent the formation of aggregates from several potentially amyloidogenic proteins or peptides, including Aβ, α-syn [109], IAPP [110], AL [111], polyglutamine (polyQ)-containing proteins, including huntingtin (htt) [112] and ATX3 [87,113].

Although the precise mechanisms by which EGCG fulfils its action differ in details depending on the different target proteins, the trait most often observed is the compound’s capability to redirect the aggregation towards off-pathway, non-toxic, β-sheet-poor aggregates, and/or remodeling the aggregates after their formation (as, for instance, in the case of htt and IAPP), rather than just retarding amyloid aggregation [112,114]. It also can interact with both monomeric protein and oligomeric aggregates.

Concerning the non-covalent interactions underlying the antiamyoid action of EGCG and related compounds, plenty of work highlights a complex pattern. In particular, hydrogen bonding with both protein backbone and hydrophilic side chains has been identified, as well as hydrophobic interactions, including those with aromatic residues [115]. This pattern points to a non-specific binding, as clearly supported by the large repertoire of proteins, both folded and disordered, EGCG can interact with.

Remarkably, EGCG was also proved to covalently bind to lysines of target proteins via Schiff base formation, which might be one factor allowing irreversible protein remodelling into non-toxic aggregates [116]. Still with regard to covalent modification EGCG can undergo, it has long been known that this molecule is subject to auto-oxidation, an issue obviously related to its bioavailability [117]. However, it has been recently reported that the oxidation products, i.e., quinone or quinonoid substances, are even more effective in preventing amyloid aggregation, quite likely by covalently binding to target protein [118].

Some literature is also available regarding the antiamyloid effects of smaller polyphenols structurally related to EGCG, in particular EGC and GA. In general, they also were proved to be effective antiamyloid agents, although to a somewhat lesser extent [113,119].

#### 2.5.2. Resveratrol

Natural sources rich in resveratrol (3,5,4′-trihydroxy-trans-stilbene, RES), are grapes, berries, red wine and other plants [120]. Several protective effects have been assigned to this compound, including antioxidant, antiinflammatory, anticarcinogenic properties, as well as a neuroprotective action in models of neurodegenerative diseases [121,122]. RES has been shown to substantially affect the amyloidogenic pathway of Aβ and IAPP. In particular, the effects on Aβ amyloid aggregation are the best characterized. This drug does not prevent oligomer formation, but retards fibril formation and even disaggregates preformed fibrils. Concomitantly, it mitigates Aβ-induced toxicity, suggestive of significant structural modifications in the oligomeric species [123]. A recent study provides structural details on the effects of RES on the mode of Aβ oligomerization [124]. In particular, it was shown that Aβ42 forms disc-shaped low molecular weight and high molecular weight oligomers (1.5–2 and 3–5 nm high, respectively), the latter resulting from the stacking of the former, and that RES prevents the stacking process, which also suggests that the largest aggregates are the most toxic ones.

The capability of this polyphenol of inhibiting the aggregation of the membrane-associated IAPP has been observed even in the presence of aggregation-fostering negatively charged lipid interfaces [125]. Based on NMR data and molecular simulations, it has been suggested that this molecule prevents ring stacking intermolecular interactions between the residues His18 and Tyr37 from adjacent polypeptide chains, quite likely via its aromatic rings [126].

#### 2.5.3. Curcumin

Curcumin ((1E,6E)-1,7-bis(4-hydroxy-3-methoxyphenyl)-1,6-heptadiene-3,5-dione) is abundantly found in the rhizomatous plant turmeric that grows in southeast Asia. It is a major component of Indian curries [127,128]. This molecule has been proved to interfere with the amyloid aggregation of Aβ [129,130,131], α-syn [132], tau [133,134] and PrP, although in this latter case there is no evidence that this interaction prevents of the appearance of toxic aggregates [135].

Overall, curcumin has been shown to prevent oligomerization, as in the case of Aβ and tau [134,136], redirect the aggregation towards nontoxic oligomers (Aβ and α-syn) [130,132] and even disaggregate preformed fibrils (Aβ, tau and α-syn) [132,134,136]. Stacking interactions between aromatic rings of the compound and aromatic residues have been implicated in the aforementioned effects, as well as hydroxy groups on the aromatic rings [137,138].

Plenty of evidence confirms that, irrespective of other well-known cytoprotective effects exerted by the molecule at the cellular level, curcumin also mitigates the neurotoxicity by directly interacting with the amyloidogenic proteins, thus preventing or reducing the appearance of the cytotoxic oligomeric forms [129,130,131,135].

Interestingly, much effort is being put into developing more potent and water-soluble curcumin analogues, solubility being a major constraint thwarting its therapeutic efficacy [129,132,133].

#### 2.5.4. Oleuropein

Oleuropein, the main phenolic compound of olive oil [139], is endowed with several beneficial effects on human health, the most prominent being anti-tumor [139], anti-inflammatory [140] and antioxidative activities [141], besides its capability to prevent the formation of toxic amyloid aggregates. This compound and its aglycone form have been proved to exert anti-amyloidogenic effects on Aβ [142], α-syn [143], β2-m [144], TTR [145], IAPP [146], and tau protein [147]. Similar to other polyphenols, the described modes of action are somewhat different depending on the protein assayed, but also share the basic features. Most often, the aggregation is redirected towards non-toxic, off-pathways. Furthermore, oleuropein displays the remarkable capacity of hindering protein binding to the plasma membrane, a key event in inducing cytotoxicity, as shown in the case of α-syn, IAPP and TTR. Interestingly, the decreased toxicity of the aggregates generated by this latter protein is likely related to the poor interaction between the resulting TTR/oleuropein aglycone complexes and monosialotetrahexosylganglioside 1 (GM1) found in the lipid rafts domains of the plasma membrane [145]. Thus, although no data are available as regards oleuropein’s functional groups involved in protein interaction, it is well established that its action on the amyloidogenic pathway results in significant cytoprotective effects.

### 2.6. Compounds Retarding Transthyretin Aggregation

In the context of the present review, a special mention deserves the class of rationally designed compounds capable of retarding TTR aggregation. This protein can cause familial forms of systemic amyloidosis in the presence of gene mutations, but also the wild type can form in the elderly amyloid deposits, mostly localized in the heart.

TTR is a homotetrameric plasma protein presenting a well defined binding site for a maximum of two tyroxines that bind the protein through a negatively cooperative manner resulting in two different affinity constants in the nanomolar and micromolar range.

In 1992, the pionieering work of Jeff Kelly [148] revealed that aggregation of TTR requires the disassembly of the tetramer into monomer and dimers and that the native tetramer is otherwise protected from the aggregation. The demonstration that tetramer disassembly, achievable in vitro under denaturing conditions, can be inhibited when the binding pockets are occupied by the natural ligand (thyroxine) or analogues have triggered an extraordinary pharmaceutical action in order to make drugable analogues of thyroxine suitable for stabilizing the TTR tetramer in vivo.

In particular, the screening of a library of substituted benzoxazoles led to the identification of tafamidis meglumine as an effective TTR stabilizer [56]. This compound is now tested in clinical trials and the early results suggest that even though a mild benefit from the treatment is achievable, there is space for improving the efficacy of this approach because a discrepancy still exists between the non physiologic in vitro model of aggregation used, so far, in drug discovery and the pathologic process occurring in vivo.

Bellotti’s group has recently discovered that disassembly of TTR and fibrillogenesis can be achieved under physiologic conditions by mechano-enzymatic mechanism consisting of a proteolytic cleavage permitted by the perturbation of the folded state in the presence of physiologic biomechanical forces [149,150]. Fibrils formed through this procedure display chemical and structural properties extremely similar to those extracted from natural deposits and highlight the role of a truncated form of TTR [149] in priming the amyloidogenesis. TTR ligands, including tafamidis, can protect TTR from the mechanoenzymatic mechanism of amyloidogenesis; however, the efficacy highly depends on the capacity of ligands to occupy both binding sites and this task is difficult to achieve in vivo due to the negative cooperativity and the low affinity of most of ligands for the second site. A good candidate for a better inhibition of the mechanoenzymatic mechanism might be Tolcapone and analogues for their property of high affinity for both binding sites and a lack of negative cooperativity. Very promising drug candidates are bivalent compounds that not only simultaneously occupy the two binding sites, but also occupy the inner channel of TTR [57].

### 2.7. Nanoparticles

Besides small molecule compounds and protein therapeutics, in recent years an increasing number of studies have focused on nanoparticles as potential inhibitors of amyloid aggregation. Nanoparticles are intriguing because they are able to cross the blood brain barrier at low concentrations, and show a certain degree of specificity towards amyloid deposits depending on their composition. In particular, while gold nanoparticles have been proved to be effective anti-aggregation molecules for insulin [151] and Aβ [152,153], polytrehalose nanoparticles have been shown to inhibit the aggregation of poly-Q proteins [154], and silver and iron oxide nanoparticles are instead capable of interfering with the aggregation of amylin [155]. Thus, despite concerns about their toxicity in some cases, nanoparticles represent a growing field, which could lead to novel anti-amyloid therapeutic approaches.

## 3. Lipid-Modulated Amyloid Aggregation and Antiamyloid Drugs

As mentioned in the introduction, it is well known that the toxicity of amyloid oligomers is largely mediated by their capability of interacting with and perturbing biological membranes [16,23]. However, further data, mostly acquired in recent times, conversely show that such interactions may also affect in several ways the mode of aggregation, thus enhancing, at least in some cases, the appearance of toxic species. Obviously, the underlying mechanisms are diverse, depending on both the protein and the membrane component involved. The interaction of specific residues of a protein with hydrophobic or charged groups in the membrane may result in unfolding and generate aggregation-prone conformations [156]. In this respect, the lipid composition plays a key role in modulating the process [157]. In particular, the interaction with negatively charged groups, such as those of anionic phospholipids, may trigger protein misfolding [158]. Furthermore, monosialotetrahexosylganglioside 1 (GM1), an abundant ganglioside that is a major component of lipid rafts, is strongly involved in favouring protein aggregation and cytotoxicity, quite likely mediated by interactions with the negatively charged sialic acid residue [159,160]. As far as cholesterol is concerned, there are contrasting reports regarding its effects on amyloid aggregation, in that it can apparently either promote or inhibit the process. Indeed, a complex picture emerges from the available data [161,162,163].

Despite the diversity of mechanisms by which membrane components stimulate amyloid aggregation, antiamyloid agents may also prevent protein/membrane interactions besides displaying classical inhibitory mechanisms of amyloid aggregation as such, which is relevant to the present review. In addition to the case of squalamine (discussed in Section 2.3.), another proof supporting this possibility was provided by experiments, whereby the interaction of α-syn with plasma membrane models was investigated in the presence or the absence of EGCG. Actually, EGCG rescued the toxicity of oligomers by reducing the flexibility of the C-terminus, which in turn completely prevented membrane permeation or disruption. Nevertheless, the flavonoid did not change the secondary structure or the size of the isolated αSN oligomers, as substantiated by solid state NMR [164]. These observations suggest that, when developing new antiamyloid drugs, their capability of interfering with protein/membrane interactions should be also carefully assessed.

## 4. The Contribution of the Computational Approaches

The high structural flexibility of many polypeptides involved in amyloid plaques formation is such that the application of molecular docking techniques to the study of ligand-target binding has generally proved to be a challenging task. In fact, docking strategies based on classical, molecular mechanics force fields usually require that the overall structure of the receptor be little influenced by the interaction with inhibitors. When this is actually the case, as for example in the functional interaction between EGCG or related lower-weight compounds (EG, EGC) and the Josephin domain—which triggers the amyloid aggregation in expanded ATX3 variants [165,166,167]—well-established docking approaches are able to provide detailed information on the structural basis of the action of inhibitors [113]. However, simulations of thermodynamic ensembles by molecular dynamics (MD)-based modeling of Aβ42 dimers, either in the presence or the absence of EGCG, showed that Aβ-EGCG interactions lead to a significant reduction in the β-content of specific regions of the peptide [168]. Similar secondary structure destabilization, accompanied by a concomitant increase in α-helix content, is common finding in MD studies of Aβ-inhibitor interaction [169,170]. Nonetheless, the structural information provided by MD simulations turned out to be a valuable starting point for extensive docking efforts, which led to the identification of key residues for the interaction with several inhibitors. Exemplary cases are the curcumin and RES interactions with Aβ peptides, which turned out to be mainly—but not exclusively—mediated by a specific stretch of peptide backbone (F19-E22) and by the side chains of two phenylalanine residues (F19 and F20) [171]. Notably, quinone derivatives (e.g., 1,4-naphthoquinon-2-yl-l-tryptophan) exert a similar mechanism of action [170]. Moreover, similar to the effects they exert on lysozyme [118], quinones are possibly able to form covalent bonds with lysine residues of Aβ peptide, which would contribute to disfavour peptides aggregation [172]. Noteworthy, in this respect, is that quinone intermediates can be formed also upon in vivo oxidation of polyphenols containing catechol residues, which may in part explain the superior inhibitory activity of some catechol-containing flavonoids [172]. Computational investigation focusing on molecules such as myricetin, quercetin and baicalein, which contain either catechol groups or adjacent dihydroxy substituents, evidenced other elements that favour inhibition. In fact, molecular docking investigations of such compounds on a tetrameric assembly of Aβ_16-21_—a relatively rigid scaffold of biochemical significance—highlighted the capability of the flavonoids under investigation of forming both polar and non-polar interactions with Lys, Phe, and Leu residues. Most importantly, the docking poses obtained indicated that their 2-phenylchromen-4-one pharmacophore plays a key role by inserting itself into the core of the Aβ_16-21_ tetramer [107].

Docking studies have been useful to also clarify the antiamyloidogenic activity of tetracyclines. As above mentioned, modelling studies based on molecular mechanics were successful in clarifying some key aspects of the mechanism of this class of inhibitors [85]. However, more recent studies have gone beyond the exploration of the conformational space of tetracyclines for the search of a pharmacophore, and aimed at the explicit modelling of protein-receptor interactions. Most notably, docking calculations on tetracycline and PrP as a receptor demonstrated that the antibiotic can specifically bind the C-terminal helix 2 of human PrP [173]. This solvent-exposed fragment of PrP is known as a potential site of nucleation toward conversion from the cellular to the pathogenic form of PrP. Such tetracycline-PrP interaction can be particularly critical because it can modulate the local geometric features of the target, which has no definite preference between α and β structure in the targeted region [173,174].

Computational studies offer the perspective to identify novel classes of inhibitors also by means of the application of virtual screening techniques on large libraries of molecular structures. Such approach requires reliable structural determination of the receptor protein as a premise. When the latter is available, ligand-receptor docking calculations making use of virtual libraries containing thousands of small molecules can lead to the identification of novel scaffolds for the development of new drugs. This kind of study was actually performed by Jiang and coworkers [175]. They used the experimentally determined structure of the Aβ_16-21_ segment after complexation with the Orange G dye as the receptor structure for a virtual screening effort on a library containing 18,000 small molecules. This allowed them to test the ability of the latter to bind the receptor efficiently, which led to the identification of a number of promising π-conjugated interactors featuring a mainly flat geometry. Subsequently, such molecules were tested on cell cultures, in order to experimentally evaluate their ability to protect cells from Aβ toxicity. The novel compounds probed in this way were actually capable of reducing the toxicity, but there was no evidence that they led to a reduction in the abundance of protein aggregates. These observations are consistent with the hypothesis that most of the toxic effects are sustained by fragments arising from the fibrils, rather than by the fibrils themselves.

Finally, it is important to underline that high-throughput computational methods were recently proved capable to efficiently screen and design peptide inhibitors against Aβ toxicity and aggregation. By using quantitative structure-activity relationship (QSAR) approaches combined with MD simulations, Wang and coworkers demonstrated that high-throughput-based strategies hold a remarkable potential for the development of peptide inhibitors sharing no sequence relationship with natural peptides [176]. By taking into account six fingerprint factors for controlling self-assembling properties of hexapeptides—i.e., bulky property, hydrophobicity, local flexibility, alpha and turn propensity, electronic properties and compositional characteristics—these authors constructed their QSAR model, training it against experimentally verified amyloidogenic databases of hexapeptides. The obtained model was used to screen and identify thousands of peptides predicted to be able to self-assemble into amyloid-like aggregates, as molecules with such a property were considered to be possibly good interactors with the Aβ peptide. A selection of the hexapeptides thus identified was further tested for the actual ability to form aggregates, using MD simulations. Then, the most promising hexapeptides were successfully probed for their inhibition activity against Aβ aggregation using biophysical experiments. Notably, in very broad terms these outcomes might also influence future developments of computational strategies devoted to the *de novo* design of anti-amyloid antibody drugs. In fact, among the most promising theoretical approaches in this context, it should be mentioned the one that focuses on the design of specific structural features of the complementarity-determining regions, the latter being relatively short sequence stretches of the antibody molecules that directly interact with the target peptides.

Recently, a complementary high-throughput method based on a quasi-structure-based drug discovery and chemical kinetic [177] has been successfully developed in order to potentiate the anti-aggregation activity of small molecules [178] towards the aggregation of Aβ. We anticipate methods of this kind, which look at the activity rather than the binding of potential inhibitor, to provide further advance of the computational design of anti-aggregation inhibitors.

The increasing level of sophistication of *in silico* analysis methods (such as for measuring the amount of β aggregates in a system) [179] and the development of approaches that combine computational and complementary experimental techniques have opened new possibilities in drug discovery and allowed the design of new types of anti-aggregation small molecules and peptides. For example, by combining molecular dynamic simulations and experimental biophysics techniques, various peptides with high affinity for amyloidogenic sequences and β-breaker properties have been identified and optimized for stability and potency [180,181].

Furthermore, thanks to multidisciplinary approaches, novel hybrid anti-aggregation molecules, which combine potential therapeutic properties from different types of small molecules and peptides have been developed, such as potent inhibitors of amyloid aggregation that consist of β-breaker motifs or compounds fused to amyloid-binding elements. In particular, quinone-tryptophan hybrid molecules [179] and endomorphin analogues conjugated to α-aminoisobutyric acid [182] have been proved to be effective in inhibiting the formation of toxic aggregates of Aβ in vitro and in cellular and fly models of disease.

## 5. Final Remarks and Perspectives

In the present review, we have discussed relevance and mechanism of action of several classes of compounds capable of contrasting amyloid aggregation. As far as low-molecular weight molecules are concerned, they can be classified into two subgroups: (i) natural compounds and (ii) synthetic molecules, the latter generally developed on the basis of drug design approaches.

As regards natural compounds, in the present review we have highlighted that they exert a wealth of beneficial effects (antioxidant, antiangiogenetic, anti-inflammatory, etc.); not only their antiamyloidogenic effect is well established, but plenty of evidence also supports the idea that this latter underlies much of the observed cytoprotective effects.

The mechanism of action of these compounds appears to be—to a certain extent—unspecific, as supported by both their capability of inhibiting the aggregation of several unrelated proteins, and by their binding affinities, in the order of micromolar. Outcomes of molecular modelling studies are in line with evidence provided by the in vitro experimentation, which indicates that rigid hydrophobic groups in active polyphenols, tetracyclines/anthracyclines and sterols play a major role in interfering with the amyloid aggregation. Also, these inhibitors generate stable patterns of hydrogen bonds with the target proteins, which are crucial in establishing a significant inhibition of the amyloidogenic pathway. In the framework of these achievements, it comes with little surprise that compounds belonging to the cited classes of inhibitors display largely superimposable effects, notwithstanding the significant structural differences they hold. In view of their cytoprotective action and their natural origin, many of the anti-amyloid compounds can be regarded as molecules to be used not only in therapy, but also in the context of amyloidosis prevention.

**Figure 3:**
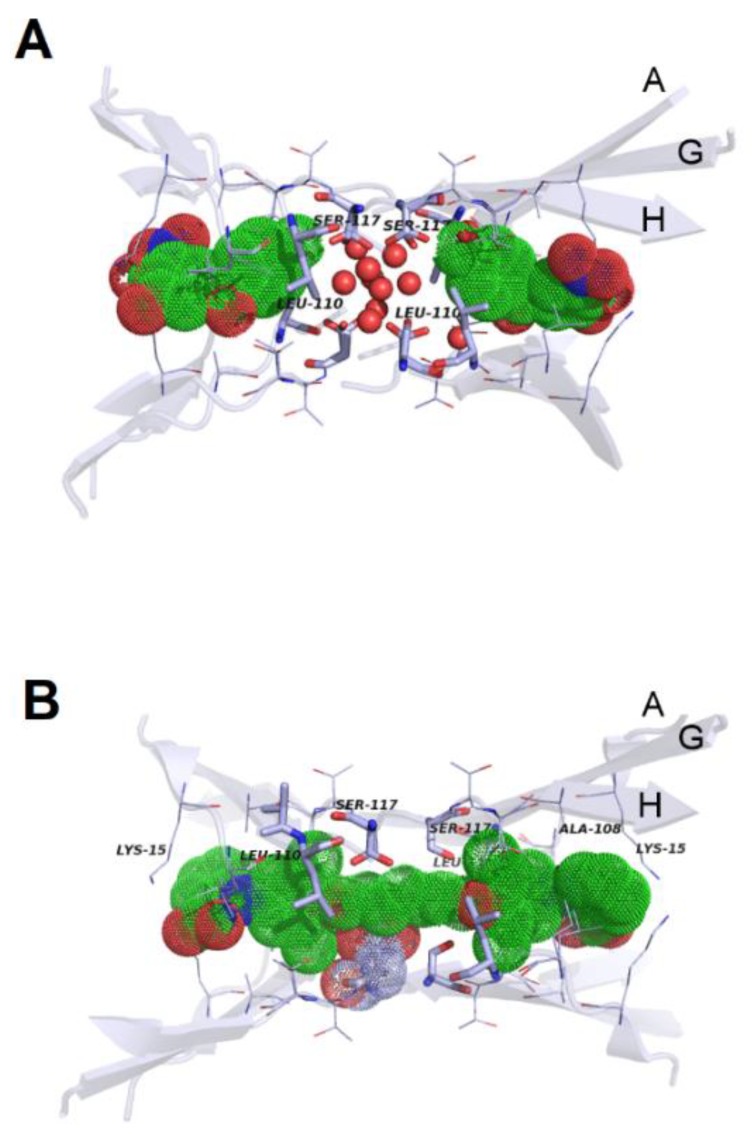

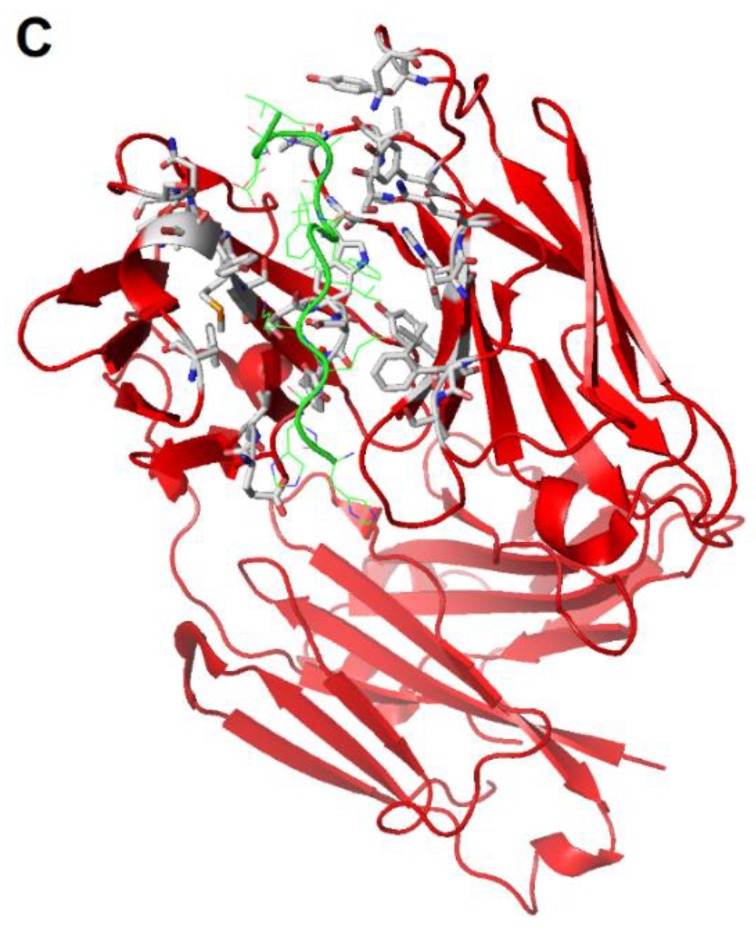
Three representative complexes of anti-amyloyd molecules with their respective targets: (**A**) TTR binding sites in the presence of tolcapone, with ligand shown as solvent accessible surface. For clarity, H_2_O oxygen atoms are shown as spheres with 50% of the van der Waals radius (PDB code 4D7B); (**B**) TTR binding sites in the presence of mds84, with ligand shown as solvent accessible surface as above (PDB code 3IPE); (**C**) crenezumab Fab in complex with Aβ, with backbones of the interactors coloured in red and green, respectively. Carbon atoms of selected side chains in the Fab are coloured in grey, whereas those belonging to Aβ are green (O, red; N, blue; S, yellow; PDB code: 5VZY). The pictorial representations in panels (**A**), (**B**) were taken from Ref. 134 and licensed under a Creative Commons Attribution 4.0. The image in panel (**C**) was created by means of PyMOL (v. 0.98), using the set of atomic coordinates available in the Protein Data Bank.

Instead, synthetic molecules are meant to be used exclusively in the case of overt amyloidoses. Design and development of such molecules can take advantage of knowledge stemming from both theoretical and experimental investigations on the mode of action of natural compounds. In particular, there is surely room for developing more effective compounds starting from the natural ones used as lead compounds. High-throughput screening studies of compounds libraries (also in the form of virtual libraries) have built on previous knowledge on inhibition mechanisms, thus disclosing new perspectives for the development of novel classes of inhibitors. Interestingly, high-throughput computational methods are expected to become increasingly useful not only in view of the development of low-molecular weight organic molecules as anti-amyloid agents, but also as a support in the efficient screening of peptide libraries for the selection of the most effective compounds against amyloid aggregation (i.e., for instance, peptide-based inhibitors derived from original amyloid sequences). Notably, this might have a significant impact also on the area of motif-grafted antibodies development, at least on the long run. Also noteworthy, in this respect, is that several 3D structures of drug/protein complexes are currently available, which quite likely will help design more and more effective molecules (some representative examples are shown in Figure 3).

In conclusion, although specificity represent at the moment a big challenge in the field of drug discovery against amyloidoses, we anticipate that the use multidisciplinary approaches that combine computational and high-throughput experimental methods on molecules of different nature could lead to anti-amyloid compounds of potential therapeutic interest and capable to inhibit in a specific manner the aggregation of amyloidogenic proteins.

## Figures and Tables

**Table 1:** A compilation of anti-aggregation compounds against amyloid diseases.

Molecule	Class	Target Protein	Disease	Key References
Squalamine *	Sterol	α-syn	PD	[35]
Trodusquemine	Sterol	α-syn	PD	[36]
Tetracycline	Tetracyclines	Prp/Aβ	APrP/AD	[37]
Doxycycline	Tetracyclines	Aβ/PrP/β2-m/TTR/LC	AD/Aβ2-m/ATTR/AL	[37,38,39,40,41,42,43,44,45]
4′Iodo-4′-doxorubicin	Anthracyclines	AL/SAA/TTR/Aβ/PrP	AD/AL/AA/ATTR/Aβ2-m	[46,47]
Acid fuchsin	Triarylmethane dye	IAPP	AD/diabetes	[48]
Fast Green FCF	Triarylmethane dye	IAPP	AD/diabetes	[49]
Crystal violet	Triarylmethine dye	tau	AD	[50]
N744	Cyanine dye	tau	AD	[51]
Congo red	Azo dye	Aβ/casein/PrP/α-syn	AD/systemic amyloidosis/prion disease/PD	[52,53]
Resveratrol	Polyphenol	Aβ/IAPP	AD/diabetes	[54]
Curcumin	Polyphenol	Aβ/tau/α-syn /htt/PrP	AD/PD/CH	[54]
EGCG	Polyphenol	Aβ/α-syn/htt/TTR/IAPP/PAP_248–286_/HEWL/k-casein and calcitonin/polyQ proteins	AD/PD/CH/HIV infectivity	[54]
Quercetin and myricetin	Polyphenol	Aβ/α-syn/insulin/IAPP	AD/PD/diabetes	[54]
Olive oil phenols	Polyphenol	Aβ/IAPP	AD/diabetes	[54]
Oleuropein	Polyphenol			
Baicalein (quinone) **	Polyphenol	tau	AD	[55]
Tafamidis (Vyndaqel)	Benzoxazole	TTR	ATTR	[56]
Tolcapone	Benzophenone	TTR	ATTR	[57]
Mds84	Palindromic ligand	TTR	ATTR	[57]
Oleocanthal **	Aldehyde	tau	AD	[55]
Cinnamaldehyde **	Aldehyde	tau	AD	[55]
Asperbenzaldehyde **	Aldehyde	tau	AD	[55]
β-Breakers	Peptide	Aβ	AD	[58]
β-Breakers	Peptide	IAPP	AD/diabetes	[59]
β-Breakers	Peptide	IAPP	AD/diabetes	[60]
β-Breakers	Peptide	Aβ/IAPP	AD/diabetes	[61]
(Bi)Cyclic peptides	Peptide	Aβ	AD	[62]
Nanobodies	Single domain antibodies	α-syn/Aβ/lysozyme/β2-m	AD/PD/systemic	[63,64,65,66]
Rationally designed antibodies	Single domain antibodies	Aβ/α-syn/IAPP	AD/PD/diabetes	[67,68,69]
Aducanumab	Monoclonal antibody	Aβ	AD	[70]
mAb158 *** (BAN2401)	Monoclonal antibody	Aβ	AD	[71]
Crenezumab	Monoclonal antibody	Aβ	AD	[72]
Gantenerumab	Monoclonal antibody	Aβ	AD	[73]
Solanezumab ****	Monoclonal antibody	Aβ	AD	[74]
Tanshinones	Diterpene	Aβ	AD	[75]
Dopamine and l-dopa	Neurotransmitter	Aβ/α-syn/IAPP	AD/PD/diabetes	[76,77]
Methylene Blue	Thiazine dye	tau/PrP/ Aβ	AD	[55,78,79]

**Notes:** * α-Syn aggregation is induced by its interaction with biological membranes. Squalamine inhibits the aggregation of α-syn by displacing it from the membranes, ** Covalent inhibitors, *** Murine version of BAN2401, **** not taken further. Information regarding the progress of clinical/preclinical trials of the compounds presented in the table is available at the following link: https://clinicaltrials.gov. Abbreviation: α-syn (α-synuclein); PrP (prion protein); Aβ (amyloid beta); APrP (Prp amyloidosis); β2-m (β2-microglobulin); Aβ2-m (β2-microglobulin amyloidosis); TTR (transthyretin); LC (Immunoglobulin light chain); ATTR (transthyretin amyloidosis); AL (immunoglobulin light chain amyloidosis); SAA (serum amyloid A); AA (serum amyloid A amyloidosis); IAPP (amylin); htt (huntingtin); CH (corea of Hungtington); HEWL (egg-white lysozyme); polyQ (polyglutamine); PAP_248–286_ (prostatic acidic phosphatase fragment); PD (Parkinson’s disease); AD (Alzheimer’s disease).

## Abbreviations

AA	serum amyloid A amyloidosis
Aβ	amyloid-beta
Aβ2-m	β2-microglobulin related amyloidosis
AD	Alzheimer’s disease
AL	immunoglobulin light chain amyloidosis
APrP	Prp amyloidosis
α-syn	alpha-synuclein
ATX3	ataxin-3
ATTR	transthyretin amyloidosis
β2-m	β2-microglobulin
CH	corea of Hungtington
CR	Congo red
cryo-EM	cryo electron microscopy
DOX	doxycycline
DRA	dialysis-related amyloidosis
EGC	(−)-epigallocatechin
EGCG	(−)-Epigallocatechin-gallate
GA	gallic acid
GM1	Monosialotetrahexosylganglioside 1
HEWL	egg-white lysozyme
htt	huntingtin
IAPP	amylin
IDOX	4′-iodo-4′-deoxy-doxorubicin
LC	immunoglobulin light chain
MD	molecular dynamics
PD	Parkinson’s disease
polyQ	polyglutamine
PrP	prion protein
RES	resveratrol (3,5,4′-trihydroxy-trans-stilbene)
QSAR	quantitative structure-activity relationship
SAA	serum amyloid A
SSNMR	solid-state nuclear magnetic resonance
TTR	transthyretin

## References

[1] Dobson C.M. (2003). Protein folding and misfolding. Nature.

[2] Hartl F.U. (2017). Protein misfolding diseases. Annu. Rev. Biochem..

[3] Chiti F., Dobson C.M. (2006). Protein misfolding, functional amyloid, and human disease. Annu. Rev. Biochem..

[4] Smith J.F., Knowles T.P., Dobson C.M., MacPhee C.E., Welland M.E. (2006). Characterization of the nanoscale properties of individual amyloid fibrils. Proc. Natl. Acad. Sci. USA.

[5] Morel B., Varela L., Conejero-Lara F. (2010). The thermodynamic stability of amyloid fibrils studied by differential scanning calorimetry. J. Phys. Chem. B.

[6] Knauer M.F., Soreghan B., Burdick D., Kosmoski J., Glabe C.G. (1992). Intracellular accumulation and resistance to degradation of the Alzheimer amyloid A4/beta protein. Proc. Natl. Acad. Sci. USA.

[7] Meier B.H., Riek R., Böckmann A. (2017). Emerging structural understanding of amyloid fibrils by solid-state NMR. Trends Biochem. Sci..

[8] Fitzpatrick A.W., Falcon B., He S., Murzin A.G., Murshudov G., Garringer H.J., Crowther R.A., Ghetti B., Goedert M., Scheres S.H. (2017). Cryo-EM structures of tau filaments from Alzheimer’s disease. Nature.

[9] Hartl F.U., Hayer-Hartl M. (2009). Converging concepts of protein folding in vitro and in vivo. Nat. Struct. Mol. Biol..

[10] Blancas-Mejía L.M., Ramirez-Alvarado M. (2013). Systemic amyloidoses. Annu. Rev. Biochem..

[11] Cohen S.I., Vendruscolo M., Dobson C.M., Knowles T.P. (2012). From macroscopic measurements to microscopic mechanisms of protein aggregation. J. Mol. Biol..

[12] Knowles T.P., Waudby C.A., Devlin G.L., Cohen S.I., Aguzzi A., Vendruscolo M., Terentjev E.M., Welland M.E., Dobson C.M. (2009). An analytical solution to the kinetics of breakable filament assembly. Science.

[13] Bucciantini M., Giannoni E., Chiti F., Baroni F., Formigli L., Zurdo J., Taddei N., Ramponi G., Dobson C.M., Stefani M. (2002). Inherent toxicity of aggregates implies a common mechanism for protein misfolding diseases. Nature.

[14] Cremades N., Cohen S.I., Deas E., Abramov A.Y., Chen A.Y., Orte A., Sandal M., Clarke R.W., Dunne P., Aprile F.A. (2012). Direct observation of the interconversion of normal and toxic forms of α-synuclein. Cell.

[15] Chen S.W., Drakulic S., Deas E., Ouberai M., Aprile F.A., Arranz R., Ness S., Roodveldt C., Guilliams T., De-Genst E.J. (2015). Structural characterization of toxic oligomers that are kinetically trapped during α-synuclein fibril formation. Proc. Natl. Acad. Sci. USA.

[16] Fusco G., Chen S.W., Williamson P.T., Cascella R., Perni M., Jarvis J.A., Cecchi C., Vendruscolo M., Chiti F., Cremades N. (2017). Structural basis of membrane disruption and cellular toxicity by α-synuclein oligomers. Science.

[17] Hsia A.Y., Masliah E., McConlogue L., Yu G.-Q., Tatsuno G., Hu K., Kholodenko D., Malenka R.C., Nicoll R.A., Mucke L. (1999). Plaque-independent disruption of neural circuits in Alzheimer’s disease mouse models. Proc. Natl. Acad. Sci. USA.

[18] Mucke L., Masliah E., Yu G.-Q., Mallory M., Rockenstein E.M., Tatsuno G., Hu K., Kholodenko D., Johnson-Wood K., McConlogue L. (2000). High-level neuronal expression of Aβ_1–42_ in wild-type human amyloid protein precursor transgenic mice: Synaptotoxicity without plaque formation. J. Neurosci..

[19] Walsh D.M., Klyubin I., Fadeeva J.V., Cullen W.K., Anwyl R., Wolfe M.S., Rowan M.J., Selkoe D.J. (2002). Naturally secreted oligomers of amyloid β protein potently inhibit hippocampal long-term potentiation in vivo. Nature.

[20] Kamenetz F., Tomita T., Hsieh H., Seabrook G., Borchelt D., Iwatsubo T., Sisodia S., Malinow R. (2003). APP processing and synaptic function. Neuron.

[21] Shankar G.M., Bloodgood B.L., Townsend M., Walsh D.M., Selkoe D.J., Sabatini B.L. (2007). Natural oligomers of the Alzheimer amyloid-β protein induce reversible synapse loss by modulating an NMDA-type glutamate receptor-dependent signaling pathway. J. Neurosci..

[22] Li S., Hong S., Shepardson N.E., Walsh D.M., Shankar G.M., Selkoe D. (2009). Soluble oligomers of amyloid β protein facilitate hippocampal long-term depression by disrupting neuronal glutamate uptake. Neuron.

[23] Flagmeier P., De S., Wirthensohn D.C., Lee S.F., Vincke C., Muyldermans S., Knowles T.P., Gandhi S., Dobson C.M., Klenerman D. (2017). Ultrasensitive measurement of Ca^2+^ influx into lipid vesicles induced by protein aggregates. Angew. Chem.-Int. Ed..

[24] Cheignon C., Tomas M., Bonnefont-Rousselot D., Faller P., Hureau C., Collin F. (2018). Oxidative stress and the amyloid beta peptide in Alzheimer’s disease. Redox Biol..

[25] Ferreira I., Bajouco L., Mota S., Auberson Y., Oliveira C., Rego A. (2012). Amyloid beta peptide 1–42 disturbs intracellular calcium homeostasis through activation of GluN2B-containing *N*-methyl-d-aspartate receptors in cortical cultures. Cell Calcium.

[26] Eckert A., Hauptmann S., Scherping I., Meinhardt J., Rhein V., Dröse S., Brandt U., Fändrich M., Müller W.E., Götz J. (2008). Oligomeric and fibrillar species of β-amyloid (Aβ42) both impair mitochondrial function in P301L tau transgenic mice. J. Mol. Med..

[27] Salminen A., Ojala J., Kauppinen A., Kaarniranta K., Suuronen T. (2009). Inflammation in Alzheimer's disease: Amyloid-β oligomers trigger innate immunity defence via pattern recognition receptors. Progr. Neurobiol..

[28] Bellotti V., Chiti F. (2008). Amyloidogenesis in its biological environment: Challenging a fundamental issue in protein misfolding diseases. Curr. Opin. Struct. Biol..

[29] Kummer M.P., Heneka M.T. (2014). Truncated and modified amyloid-beta species. Alzheimer Res. Ther..

[30] Galvagnion C., Buell A.K., Meisl G., Michaels T.C., Vendruscolo M., Knowles T.P., Dobson C.M. (2015). Lipid vesicles trigger α-synuclein aggregation by stimulating primary nucleation. Nat. Chem. Biol..

[31] Merlini G., Bellotti V. (2003). Molecular mechanisms of amyloidosis. N. Engl. J. Med..

[32] Stoppini M., Bellotti V. (2015). Systemic amyloidosis: lessons from β2-microglobulin. J. Biol. Chem..

[33] Valleix S., Gillmore J.D., Bridoux F., Mangione P.P., Dogan A., Nedelec B., Boimard M., Touchard G., Goujon J.-M., Lacombe C. (2012). Hereditary systemic amyloidosis due to Asp76Asn variant β2-microglobulin. N. Engl. J. Med..

[34] Marcoux J., Mangione P.P., Porcari R., Degiacomi M.T., Verona G., Taylor G.W., Giorgetti S., Raimondi S., Sanglier-Cianférani S., Benesch J.L. (2015). A novel mechano-enzymatic cleavage mechanism underlies transthyretin amyloidogenesis. EMBO Mol. Med..

[35] Perni M., Galvagnion C., Maltsev A., Meisl G., Müller M.B., Challa P.K., Kirkegaard J.B., Flagmeier P., Cohen S.I., Cascella R. (2017). A natural product inhibits the initiation of α-synuclein aggregation and suppresses its toxicity. Proc. Natl. Acad. Sci. USA.

[36] Perni M., Flagmeier P., Limbocker R., Cascella R., Aprile F.A., Galvagnion C., Heller G.T., Meisl G., Chen S.W., Kumita J.R. (2018). Multistep inhibition of α-synuclein aggregation and toxicity in vitro and in vivo by trodusquemine. ACS Chem. Biol..

[37] Forloni G., Colombo L., Girola L., Tagliavini F., Salmona M. (2001). Anti-amyloidogenic activity of tetracyclines: Studies in vitro. FEBS Lett..

[38] Airoldi C., Colombo L., Manzoni C., Sironi E., Natalello A., Doglia S.M., Forloni G., Tagliavini F., Del Favero E., Cantu L. (2011). Tetracycline prevents Aβ oligomer toxicity through an atypical supramolecular interaction. Org. Biomol. Chem..

[39] Liu T., Marcinko T.M., Kiefer P.A., Vachet R.W. (2017). Using covalent labeling and mass spectrometry to study protein binding sites of amyloid inhibiting molecules. Anal. Chem..

[40] Giorgetti S., Raimondi S., Pagano K., Relini A., Bucciantini M., Corazza A., Fogolari F., Codutti L., Salmona M., Mangione P. (2011). Effect of tetracyclines on the dynamics of formation and destructuration of β2-microglobulin amyloid fibrils. J. Biol. Chem..

[41] Marcinko T.M., Dong J., LeBlanc R., Daborowski K.V., Vachet R.W. (2017). Small molecule-mediated inhibition of β-2-microglobulin amyloid fibril formation. J. Biol. Chem..

[42] Obici L., Cortese A., Lozza A., Lucchetti J., Gobbi M., Palladini G., Perlini S., Saraiva M.J., Merlini G. (2012). Doxycycline plus tauroursodeoxycholic acid for transthyretin amyloidosis: a phase II study. Amyloid.

[43] Montagna G., Cazzulani B., Obici L., Uggetti C., Giorgetti S., Porcari R., Ruggiero R., Mangione P.P., Brambilla M., Lucchetti J. (2013). Benefit of doxycycline treatment on articular disability caused by dialysis related amyloidosis. Amyloid.

[44] Piccoli G.B., Hachemi M., Molfino I., Coindre J.P., Boursot C. (2017). Doxycycline treatment in dialysis related amyloidosis: Discrepancy between antalgic effect and inflammation, studied with FDG-positron emission tomography: A case report. BMC Nephrol..

[45] Wechalekar A., Whelan C. (2017). Encouraging impact of doxycycline on early mortality in cardiac light chain (AL) amyloidosis. Blood Cancer J..

[46] Gianni L., Bellotti V., Gianni A.M., Merlini G. (1995). New drug therapy of amyloidoses: resorption of AL-type deposits with 4′-iodo-4′-deoxydoxorubicin. Blood.

[47] Merlini G., Ascari E., Amboldi N., Bellotti V., Arbustini E., Perfetti V., Ferrari M., Zorzoli I., Marinone M.G., Garini P. (1995). Interaction of the anthracycline 4′-iodo-4′-deoxydoxorubicin with amyloid fibrils: Inhibition of amyloidogenesis. Proc. Natl. Acad. Sci. USA.

[48] Meng F., Abedini A., Plesner A., Middleton C.T., Potter K.J., Zanni M.T., Verchere C.B., Raleigh D.P. (2010). The sulfated triphenyl methane derivative acid fuchsin is a potent inhibitor of amyloid formation by human islet amyloid polypeptide and protects against the toxic effects of amyloid formation. J. Mol. Biol..

[49] How S.C., Yang S.M., Hsin A., Tseng C.P., Hsueh S.S., Lin M.S., Chen R.P., Chou W.L., Wang S.S. (2016). Examining the inhibitory potency of food additive fast green FCF against amyloid fibrillogenesis under acidic conditions. Food Funct..

[50] Chang E., Congdon E.E., Honson N.S., Duff K.E., Kuret J. (2009). Structure–activity relationship of cyanine tau aggregation inhibitors. J. Med. Chem..

[51] Necula M., Chirita C.N., Kuret J. (2005). Cyanine dye N744 inhibits tau fibrillization by blocking filament extension: Implications for the treatment of tauopathic neurodegenerative diseases. Biochemistry.

[52] Frid P., Anisimov S.V., Popovic N. (2007). Congo red and protein aggregation in neurodegenerative diseases. Brain Res. Rev..

[53] Lendel C., Bolognesi B., Wahlström A., Dobson C.M., Gräslund A. (2010). Detergent-like interaction of Congo red with the amyloid β peptide. Biochemistry.

[54] Stefani M., Rigacci S. (2013). Protein folding and aggregation into amyloid: the interference by natural phenolic compounds. Int. J. Mol. Sci..

[55] Cisek K., Cooper G.L., Huseby C.J., Kuret J. (2014). Structure and mechanism of action of tau aggregation inhibitors. Curr. Alzheimer Res..

[56] Razavi H., Palaninathan S.K., Powers E.T., Wiseman R.L., Purkey H.E., Mohamedmohaideen N.N., Deechongkit S., Chiang K.P., Dendle M.T., Sacchettini J.C. (2003). Benzoxazoles as transthyretin amyloid fibril inhibitors: Synthesis, evaluation, and mechanism of action. Angew. Chem.-Int. Ed..

[57] Verona G., Mangione P.P., Raimondi S., Giorgetti S., Faravelli G., Porcari R., Corazza A., Gillmore J.D., Hawkins P.N., Pepys M.B. (2017). Inhibition of the mechano-enzymatic amyloidogenesis of transthyretin: role of ligand affinity, binding cooperativity and occupancy of the inner channel. Sci. Rep..

[58] Sievers S.A., Karanicolas J., Chang H.W., Zhao A., Jiang L., Zirafi O., Stevens J.T., Münch J., Baker D., Eisenberg D. (2011). Structure-based design of non-natural amino-acid inhibitors of amyloid fibril formation. Nature.

[59] Sivanesam K., Shu I., Huggins K.N., Tatarek-Nossol M., Kapurniotu A., Andersen N.H. (2016). Peptide inhibitors of the amyloidogenesis of IAPP: Verification of the hairpin-binding geometry hypothesis. FEBS Lett..

[60] Andreetto E., Malideli E., Yan L.M., Kracklauer M., Farbiarz K., Tatarek-Nossol M., Rammes G., Prade E., Neumüller T., Caporale A. (2015). A hot-segment-based approach for the design of cross-amyloid interaction surface mimics as inhibitors of amyloid self-assembly. Angew. Chem.-Int. Ed..

[61] Yan L.M., Velkova A., Tatarek-Nossol M., Rammes G., Sibaev A., Andreetto E., Kracklauer M., Bakou M., Malideli E., Göke B. (2013). Selectively *N*-methylated soluble IAPP mimics as potent IAPP receptor agonists and nanomolar inhibitors of cytotoxic self-assembly of both IAPP and Aβ40. Angew. Chem.-Int. Ed..

[62] Truex N.L., Wang Y., Nowick J.S. (2016). Assembly of peptides derived from β-sheet regions of β-amyloid. J. Am. Chem. Soc..

[63] Chan P.-H., Pardon E., Menzer L., De Genst E., Kumita J.R., Christodoulou J., Saerens D., Brans A., Bouillenne F., Archer D.B. (2008). Engineering a camelid antibody fragment that binds to the active site of human lysozyme and inhibits its conversion into amyloid fibrils. Biochemistry.

[64] El-Turk F., Newby F.N., De Genst E., Guilliams T., Sprules T., Mittermaier A., Dobson C.M., Vendruscolo M. (2016). Structural effects of two camelid nanobodies directed to distinct C-terminal epitopes on α-synuclein. Biochemistry.

[65] Drews A., Flint J., Shivji N., Jönsson P., Wirthensohn D., De Genst E., Vincke C., Muyldermans S., Dobson C., Klenerman D. (2016). Individual aggregates of amyloid beta induce temporary calcium influx through the cell membrane of neuronal cells. Sci. Rep..

[66] Raimondi S., Porcari R., Mangione P.P., Verona G., Marcoux J., Giorgetti S., Taylor G.W., Ellmerich S., Ballico M., Zanini S. (2017). A specific nanobody prevents amyloidogenesis of D76N β 2-microglobulin in vitro and modifies its tissue distribution in vivo. Sci. Rep..

[67] Perchiacca J.M., Ladiwala A.R.A., Bhattacharya M., Tessier P.M. (2012). Structure-based design of conformation-and sequence-specific antibodies against amyloid β. Proc. Natl. Acad. Sci. USA.

[68] Sormanni P., Aprile F.A., Vendruscolo M. (2015). Rational design of antibodies targeting specific epitopes within intrinsically disordered proteins. Proc. Natl. Acad. Sci. USA.

[69] Aprile F.A., Sormanni P., Perni M., Arosio P., Linse S., Knowles T.P., Dobson C.M., Vendruscolo M. (2017). Selective targeting of primary and secondary nucleation pathways in Aβ42 aggregation using a rational antibody scanning method. Sci. Adv..

[70] Sevigny J., Chiao P., Bussière T., Weinreb P.H., Williams L., Maier M., Dunstan R., Salloway S., Chen T., Ling Y. (2016). The antibody aducanumab reduces Aβ plaques in Alzheimer’s disease. Nature.

[71] Tucker S., Möller C., Tegerstedt K., Lord A., Laudon H., Sjödahl J., Söderberg L., Spens E., Sahlin C., Waara E.R. (2015). The murine version of BAN2401 (mAb158) selectively reduces amyloid-β protofibrils in brain and cerebrospinal fluid of tg-ArcSwe mice. J. Alzheimers Dis..

[72] Adolfsson O., Pihlgren M., Toni N., Varisco Y., Buccarello A.L., Antoniello K., Lohmann S., Piorkowska K., Gafner V., Atwal J.K. (2012). An effector-reduced anti-β-amyloid (Aβ) antibody with unique aβ binding properties promotes neuroprotection and glial engulfment of Aβ. J. Neurosci..

[73] Bohrmann B., Baumann K., Benz J., Gerber F., Huber W., Knoflach F., Messer J., Oroszlan K., Rauchenberger R., Richter W.F. (2012). Gantenerumab: A novel human anti-Aβ antibody demonstrates sustained cerebral amyloid-β binding and elicits cell-mediated removal of human amyloid-β. J. Alzheimers Dis..

[74] Lannfelt L., Relkin N.R., Siemers E.R. (2014). Amyloid-β-directed immunotherapy for Alzheimer's disease. J. Intern. Med..

[75] Wang Q., Yu X., Patal K., Hu R., Chuang S., Zhang G., Zheng J. (2013). Tanshinones inhibit amyloid aggregation by amyloid-β peptide, disaggregate amyloid fibrils, and protect cultured cells. ACS Chem. Neurosci..

[76] Li J., Zhu M., Manning-Bog A.B., Di Monte D.A., Fink A.L. (2004). Dopamine and L-dopa disaggregate amyloid fibrils: Implications for Parkinson's and Alzheimer's disease. FASEB J..

[77] Saunders J.C., Young L.M., Mahood R.A., Jackson M.P., Revill C.H., Foster R.J., Smith D.A., Ashcroft A.E., Brockwell D.J., Radford S.E. (2016). An in vivo platform for identifying inhibitors of protein aggregation. Nat. Chem. Biol..

[78] Cavaliere P., Torrent J., Prigent S., Granata V., Pauwels K., Pastore A., Rezaei H., Zagari A. (2013). Binding of methylene blue to a surface cleft inhibits the oligomerization and fibrillization of prion protein. Biochim. Biophys. Acta.

[79] Necula M., Breydo L., Milton S., Kayed R., van der Veer W.E., Tone P., Glabe C.G. (2007). Methylene blue inhibits amyloid Aβ oligomerization by promoting fibrillization. Biochemistry.

[80] Heller G.T., Aprile F.A., Vendruscolo M. (2017). Methods of probing the interactions between small molecules and disordered proteins. Cell. Mol. Life Sci..

[81] Younan N.D., Viles J.H. (2015). A comparison of three fluorophores for the detection of amyloid fibers and prefibrillar oligomeric assemblies. ThT (thioflavin T); ANS (1-anilinonaphthalene-8-sulfonic acid); and bisANS (4, 4′-dianilino-1, 1′-binaphthyl-5, 5′-disulfonic acid). Biochemistry.

[82] Qin L., Vastl J., Gao J. (2010). Highly sensitive amyloid detection enabled by thioflavin T dimers. Mol. Biosyst..

[83] Nesterov E.E., Skoch J., Hyman B.T., Klunk W.E., Bacskai B.J., Swager T.M. (2005). In vivo optical imaging of amyloid aggregates in brain: Design of fluorescent markers. Angew. Chem.-Int. Ed..

[84] Tagliavini F., Forloni G., Colombo L., Rossi G., Girola L., Canciani B., Angeretti N., Giampaolo L., Peressini E., Awan T. (2000). Tetracycline affects abnormal properties of synthetic PrP peptides and PrPSc in vitro. J. Mol. Biol..

[85] Cosentino U., Varí M.R., Saracino A.G., Pitea D., Moro G., Salmona M. (2005). Tetracycline and its analogues as inhibitors of amyloid fibrils: searching for a geometrical pharmacophore by theoretical investigation of their conformational behavior in aqueous solution. J. Mol. Model..

[86] Eisenberg D.S., Sawaya M.R. (2017). Structural studies of amyloid proteins at the molecular level. Annu. Rev. Biochem..

[87] Bonanomi M., Natalello A., Visentin C., Pastori V., Penco A., Cornelli G., Colombo G., Malabarba M.G., Doglia S.M., Relini A. (2014). Epigallocatechin-3-gallate and tetracycline differently affect ataxin-3 fibrillogenesis and reduce toxicity in spinocerebellar ataxia type 3 model. Hum. Mol. Genet..

[88] Bonanomi M., Visentin C., Natalello A., Spinelli M., Vanoni M., Airoldi C., Regonesi M.E., Tortora P. (2015). How epigallocatechin-3-gallate and tetracycline interact with the josephin domain of ataxin-3 and alter its aggregation mode. Chem.-Eur. J..

[89] Rao M.N., Shinnar A.E., Noecker L.A., Chao T.L., Feibush B., Snyder B., Sharkansky I., Sarkahian A., Zhang X., Jones S.R. (2000). Aminosterols from the dogfish shark *Squalus acanthias*. J. Nat. Prod..

[90] Sills A.K., Williams J.I., Tyler B.M., Epstein D.S., Sipos E.P., Davis J.D., McLane M.P., Pitchford S., Cheshire K., Gannon F.H. (1998). Squalamine inhibits angiogenesis and solid tumor growth in vivo and perturbs embryonic vasculature. Cancer Res..

[91] Spillantini M.G., Schmidt M.L., Lee V.M.-Y., Trojanowski J.Q., Jakes R., Goedert M. (1997). α-Synuclein in lewy bodies. Nature.

[92] Young L.M., Ashcroft A.E., Radford S.E. (2017). Small molecule probes of protein aggregation. Curr. Opin. Chem. Biol..

[93] Viet M.H., Ngo S.T., Lam N.S., Li M.S. (2011). Inhibition of aggregation of amyloid peptides by beta-sheet breaker peptides and their binding affinity. J. Phys. Chem. B.

[94] Soto C., Sigurdsson E.M., Morelli L., Kumar R.A., Castaño E.M., Frangione B. (1998). β-sheet breaker peptides inhibit fibrillogenesis in a rat brain model of amyloidosis: Implications for Alzheimer's therapy. Nat. Med..

[95] Poduslo J.F., Curran G.L., Kumar A., Frangione B., Soto C. (1999). β-Sheet breaker peptide inhibitor of Alzheimer's amyloidogenesis with increased blood–brain barrier permeability and resistance to proteolytic degradation in plasma. J. Neurobiol..

[96] Adessi C., Soto C. (2002). Converting a peptide into a drug: strategies to improve stability and bioavailability. Curr. Med. Chem..

[97] Jha A., Kumar M.G., Gopi H.N., Paknikar K.M. (2018). Inhibition of β-amyloid aggregation through a designed β-hairpin peptide. Langmuir.

[98] Hoyer W., Grönwall C., Jonsson A., Ståhl S., Härd T. (2008). Stabilization of a β-hairpin in monomeric Alzheimer's amyloid-β peptide inhibits amyloid formation. Proc. Natl. Acad. Sci. USA.

[99] Mirecka E.A., Shaykhalishahi H., Gauhar A., Akgül Ş., Lecher J., Willbold D., Stoldt M., Hoyer W. (2014). Sequestration of a β-hairpin for control of α-synuclein aggregation. Angew. Chem.-Int. Ed..

[100] Shaykhalishahi H., Mirecka E.A., Gauhar A., Grüning C.S., Willbold D., Härd T., Stoldt M., Hoyer W. (2015). A β-Hairpin-Binding protein for three different disease-related amyloidogenic proteins. ChemBioChem.

[101] Castillo-Carranza D.L., Sengupta U., Guerrero-Muñoz M.J., Lasagna-Reeves C.A., Gerson J.E., Singh G., Estes D.M., Barrett A.D., Dineley K.T., Jackson G.R. (2014). Passive immunization with Tau oligomer monoclonal antibody reverses tauopathy phenotypes without affecting hyperphosphorylated neurofibrillary tangles. J. Neurosci..

[102] Guilliams T., El-Turk F., Buell A.K., O'Day E.M., Aprile F.A., Esbjörner E.K., Vendruscolo M., Cremades N., Pardon E., Wyns L. (2013). Nanobodies raised against monomeric α-synuclein distinguish between fibrils at different maturation stages. J. Mol. Biol..

[103] Arosio P., Michaels T.C., Linse S., Månsson C., Emanuelsson C., Presto J., Johansson J., Vendruscolo M., Dobson C.M., Knowles T.P. (2016). Kinetic analysis reveals the diversity of microscopic mechanisms through which molecular chaperones suppress amyloid formation. Nat. Commun..

[104] Aprile F.A., Arosio P., Fusco G., Chen S.W., Kumita J.R., Dhulesia A., Tortora P., Knowles T.P., Vendruscolo M., Dobson C.M. (2017). Inhibition of α-synuclein fibril elongation by Hsp70 is governed by a kinetic binding competition between α-synuclein species. Biochemistry.

[105] Aprile F.A., Sormanni P., Vendruscolo M. (2015). A rational design strategy for the selective activity enhancement of a molecular chaperone toward a target substrate. Biochemistry.

[106] Bongiovanni M.N., Aprile F.A., Sormanni P., Vendruscolo M. (2018). A rationally designed Hsp70 variant rescues the aggregation-associated toxicity of human IAPP in cultured pancreatic islet β-cells. Int. J. Mol. Sci..

[107] Bu X.-L., Rao P.P., Wang Y.-J. (2016). Anti-amyloid aggregation activity of natural compounds: Implications for Alzheimer’s drug discovery. Mol. Neurobiol..

[108] Liu Y., Pukala T.L., Musgrave I.F., Williams D.M., Dehle F.C., Carver J.A. (2013). Gallic acid is the major component of grape seed extract that inhibits amyloid fibril formation. Bioorg. Med. Chem. Lett..

[109] Yang J.E., Rhoo K.Y., Lee S., Lee J.T., Park J.H., Bhak G., Paik S.R. (2017). EGCG-mediated protection of the membrane disruption and cytotoxicity caused by the ‘active Oligomer’of α-synuclein. Sci. Rep..

[110] Young L.M., Cao P., Raleigh D.P., Ashcroft A.E., Radford S.E. (2014). Ion mobility spectrometry–mass spectrometry defines the oligomeric intermediates in amylin amyloid formation and the mode of action of inhibitors. J. Am. Chem. Soc..

[111] Andrich K., Hegenbart U., Kimmich C., Kedia N., Bergen H.R., Schönland S., Wanker E.E., Bieschke J. (2017). Aggregation of full-length immunoglobulin light chains from systemic light chain amyloidosis (AL) patients is remodeled by epigallocatechin-3-gallate. J. Biol. Chem..

[112] Ehrnhoefer D.E., Bieschke J., Boeddrich A., Herbst M., Masino L., Lurz R., Engemann S., Pastore A., Wanker E.E. (2008). EGCG redirects amyloidogenic polypeptides into unstructured, off-pathway oligomers. Nat. Struct. Mol. Biol..

[113] Visentin C., Pellistri F., Natalello A., Vertemara J., Bonanomi M., Gatta E., Penco A., Relini A., De Gioia L., Airoldi C. (2017). Epigallocatechin-3-gallate and related phenol compounds redirect the amyloidogenic aggregation pathway of ataxin-3 towards non-toxic aggregates and prevent toxicity in neural cells and *Caenorhabditis elegans* animal model. Hum. Mol. Genet..

[114] Mo Y., Lei J., Sun Y., Zhang Q., Wei G. (2016). Conformational ensemble of hIAPP dimer: insight into the molecular mechanism by which a green tea extract inhibits hIAPP aggregation. Sci. Rep..

[115] Wang S.-H., Dong X.-Y., Sun Y. (2012). Thermodynamic analysis of the molecular interactions between amyloid β-protein fragments and (−)-epigallocatechin-3-gallate. J. Phys. Chem. B.

[116] Palhano F.L., Lee J., Grimster N.P., Kelly J.W. (2013). Toward the molecular mechanism (s) by which EGCG treatment remodels mature amyloid fibrils. J. Am Chem. Soc..

[117] Severino J.F., Goodman B.A., Kay C.W., Stolze K., Tunega D., Reichenauer T.G., Pirker K.F. (2009). Free radicals generated during oxidation of green tea polyphenols: electron paramagnetic resonance spectroscopy combined with density functional theory calculations. Free Radic. Biol. Med..

[118] An T.-T., Feng S., Zeng C.-M. (2017). Oxidized epigallocatechin gallate inhibited lysozyme fibrillation more strongly than the native form. Redox Biol..

[119] Liu Y., Carver J.A., Calabrese A.N., Pukala T.L. (2014). Gallic acid interacts with α-synuclein to prevent the structural collapse necessary for its aggregation. Biochim. Biophys. Acta.

[120] Jia Y., Liu Z., Huo X., Wang C., Meng Q., Liu Q., Sun H., Sun P., Yang X., Shu X. (2015). Enhancement effect of resveratrol on the intestinal absorption of bestatin by regulating PEPT1, MDR1 and MRP2 in vivo and in vitro. Int. J. Pharm..

[121] Pallàs M., Porquet D., Vicente A., Sanfeliu C. (2013). Resveratrol: New avenues for a natural compound in neuroprotection. Curr. Pharm. Des..

[122] Lopez-Miranda V., Soto-Montenegro M., Vera G., Herradon E., Desco M., Abalo R. (2012). Resveratrol: A neuroprotective polyphenol in the mediterranean diet. Rev. Neurol..

[123] Feng Y., Wang X.-P., Yang S.-G., Wang Y.-J., Zhang X., Du X.-T., Sun X.-X., Zhao M., Huang L., Liu R.-T. (2009). Resveratrol inhibits beta-amyloid oligomeric cytotoxicity but does not prevent oligomer formation. Neurotoxicology.

[124] Fu Z., Aucoin D., Ahmed M., Ziliox M., Van Nostrand W.E., Smith S.O. (2014). Capping of Aβ42 oligomers by small molecule inhibitors. Biochemistry.

[125] Evers F., Jeworrek C., Tiemeyer S., Weise K., Sellin D., Paulus M., Struth B., Tolan M., Winter R. (2009). Elucidating the mechanism of lipid membrane-induced IAPP fibrillogenesis and its inhibition by the red wine compound resveratrol: A synchrotron X-ray reflectivity study. J. Am. Chem. Soc..

[126] Wei L., Jiang P., Xu W., Li H., Zhang H., Yan L., Chan-Park M.B., Liu X.-W., Tang K., Mu Y. (2011). The molecular basis of distinct aggregation pathways of islet amyloid polypeptide. J. Biol. Chem..

[127] Monroy A., Lithgow G.J., Alavez S. (2013). Curcumin and neurodegenerative diseases. Biofactors.

[128] Hatcher H., Planalp R., Cho J., Torti F., Torti S. (2008). Curcumin: from ancient medicine to current clinical trials. Cell. Mol. Life Sci..

[129] Endo H., Nikaido Y., Nakadate M., Ise S., Konno H. (2014). Structure activity relationship study of curcumin analogues toward the amyloid-beta aggregation inhibitor. Bioorg. Med. Chem. Lett..

[130] Thapa A., Jett S.D., Chi E.Y. (2016). Curcumin attenuates amyloid-β aggregate toxicity and modulates amyloid-β aggregation pathway. ACS Chem. Neurosci..

[131] Liang F., Wan Y., Schaak D., Ward J., Shen X., Tanzi R.E., Zhang C., Quan Q. (2017). Nanoplasmonic fiber tip probe detects significant reduction of intracellular Alzheimer’s disease-related oligomers by curcumin. Sci. Rep..

[132] Ahsan N., Mishra S., Jain M.K., Surolia A., Gupta S. (2015). Curcumin Pyrazole and its derivative (*N*-(3-Nitrophenylpyrazole) curcumin inhibit aggregation, disrupt fibrils and modulate toxicity of wild type and mutant α-synuclein. Sci. Rep..

[133] Okuda M., Fujita Y., Hijikuro I., Wada M., Uemura T., Kobayashi Y., Waku T., Tanaka N., Nishimoto T., Izumi Y. (2017). PE859, A novel curcumin derivative, inhibits amyloid-β and Tau aggregation, and ameliorates cognitive dysfunction in senescence-accelerated mouse prone 8. J. Alzheimers Dis..

[134] Rane J.S., Bhaumik P., Panda D. (2017). Curcumin inhibits tau aggregation and disintegrates preformed tau filaments in vitro. J. Alzheimer Dis..

[135] Hafner-Bratkovič I., Gašperšič J., Šmid L.M., Bresjanac M., Jerala R. (2008). Curcumin binds to the α-helical intermediate and to the amyloid form of prion protein—A new mechanism for the inhibition of PrPSc accumulation. J. Neurochem..

[136] Yang F., Lim G.P., Begum A.N., Ubeda O.J., Simmons M.R., Ambegaokar S.S., Chen P.P., Kayed R., Glabe C.G., Frautschy S.A. (2005). Curcumin inhibits formation of amyloid β oligomers and fibrils, binds plaques, and reduces amyloid in vivo. J. Biol. Chem..

[137] Yanagisawa D., Taguchi H., Morikawa S., Kato T., Hirao K., Shirai N., Tooyama I. (2015). Novel curcumin derivatives as potent inhibitors of amyloid β aggregation. Biochem. Biophys. Rep..

[138] Orteca G., Tavanti F., Bednarikova Z., Gazova Z., Rigillo G., Imbriano C., Basile V., Asti M., Rigamonti L., Saladini M. (2018). Curcumin derivatives and Aβ-fibrillar aggregates: An interactions’ study for diagnostic/therapeutic purposes in neurodegenerative diseases. Bioorg. Med. Chem..

[139] Sherif I.O., Al-Gayyar M.M. (2018). Oleuropein potentiates anti-tumor activity of cisplatin against HepG2 through affecting proNGF/NGF balance. Life Sci..

[140] Qabaha K., Al-Rimawi F., Qasem A., Naser S.A. (2018). Oleuropein is responsible for the major anti-inflammatory effects of olive leaf extract. J. Med. Food..

[141] Umeno A., Takashima M., Murotomi K., Nakajima Y., Koike T., Matsuo T., Yoshida Y. (2015). Radical-scavenging activity and antioxidative effects of olive leaf components oleuropein and hydroxytyrosol in comparison with homovanillic alcohol. J. Oleo Sci..

[142] Rigacci S., Guidotti V., Bucciantini M., Nichino D., Relini A., Berti A., Stefani M. (2011). Aβ (1–42) aggregates into non-toxic amyloid assemblies in the presence of the natural polyphenol oleuropein aglycon. Curr. Alzheimer Res..

[143] Palazzi L., Bruzzone E., Bisello G., Leri M., Stefani M., Bucciantini M., Polverino de Laureto P. (2018). Oleuropein aglycone stabilizes the monomeric α-synuclein and favours the growth of non-toxic aggregates. Sci. Rep..

[144] Leri M., Oropesa-Nuñez R., Canale C., Raimondi S., Giorgetti S., Bruzzone E., Bellotti V., Stefani M., Bucciantini M. (2018). Oleuropein aglycone: A polyphenol with different targets against amyloid toxicity. Biochim. Biophys. Acta.

[145] Leri M., Nosi D., Natalello A., Porcari R., Ramazzotti M., Chiti F., Bellotti V., Doglia S.M., Stefani M., Bucciantini M. (2016). The polyphenol oleuropein aglycone hinders the growth of toxic transthyretin amyloid assemblies. J. Nutr. Biochem..

[146] Rigacci S., Guidotti V., Bucciantini M., Parri M., Nediani C., Cerbai E., Stefani M., Berti A. (2010). Oleuropein aglycon prevents cytotoxic amyloid aggregation of human amylin. J. Nutr. Biochem..

[147] Daccache A., Lion C., Sibille N., Gerard M., Slomianny C., Lippens G., Cotelle P. (2011). Oleuropein and derivatives from olives as Tau aggregation inhibitors. Neurochem. Int..

[148] Colon W., Kelly J.W. (1992). Partial denaturation of transthyretin is sufficient for amyloid fibril formation in vitro. Biochemistry.

[149] Mangione P.P., Porcari R., Gillmore J.D., Pucci P., Monti M., Porcari M., Giorgetti S., Marchese L., Raimondi S., Serpell L.C. (2014). Proteolytic cleavage of Ser52Pro variant transthyretin triggers its amyloid fibrillogenesis. Proc. Natl. Acad. Sci. USA.

[150] Mangione P.P., Verona G., Corazza A., Marcoux J., Canetti D., Giorgetti S., Raimondi S., Stoppini M., Esposito M., Relini A. (2018). Plasminogen activation triggers transthyretin amyloidogenesis in vitro. J. Biol. Chem..

[151] Hsieh S., Chang C.-W., Chou H.-H. (2013). Gold nanoparticles as amyloid-like fibrillogenesis inhibitors. Colloids Surf. B Biointerfaces.

[152] Liao Y.H., Chang Y.J., Yoshiike Y., Chang Y.C., Chen Y.R. (2012). Negatively charged gold nanoparticles inhibit Alzheimer's amyloid-β fibrillization, induce fibril dissociation, and mitigate neurotoxicity. Small.

[153] Song M., Sun Y., Luo Y., Zhu Y., Liu Y., Li H. (2018). Exploring the mechanism of inhibition of Au nanoparticles on the aggregation of amyloid-β (16-22) peptides at the atom level by all-atom molecular dynamics. Int. J. Mol. Sci..

[154] Debnath K., Pradhan N., Singh B.K., Jana N.R. (2017). Poly(trehalose) nanoparticles prevent amyloid aggregation and suppress polyglutamine aggregation in a Huntington’s Disease model mouse. ACS Appl. Mater. Interfaces.

[155] Wang M., Kakinen A., Pilkington E.H., Davis T.P., Ke P.C. (2017). Differential effects of silver and iron oxide nanoparticles on IAPP amyloid aggregation. Biomater. Sci..

[156] Bokvist M., Lindström F., Watts A., Gröbner G. (2004). Two types of Alzheimer's β-amyloid (1–40) peptide membrane interactions: Aggregation preventing transmembrane anchoring versus accelerated surface fibril formation. J. Mol. Biol..

[157] Galvagnion C., Brown J.W., Ouberai M.M., Flagmeier P., Vendruscolo M., Buell A.K., Sparr E., Dobson C.M. (2016). Chemical properties of lipids strongly affect the kinetics of the membrane-induced aggregation of α-synuclein. Proc. Natl. Acad. Sci. USA.

[158] Al Kayal T., Russo E., Pieri L., Caminati G., Berti D., Bucciantini M., Stefani M., Baglioni P. (2012). Interactions of lysozyme with phospholipid vesicles: effects of vesicle biophysical features on protein misfolding and aggregation. Soft Matter.

[159] Matsuzaki K., Kato K., Yanagisawa K. (2010). Aβ polymerization through interaction with membrane gangliosides. Biochim. Biophys. Acta.

[160] Bucciantini M., Nosi D., Forzan M., Russo E., Calamai M., Pieri L., Formigli L., Quercioli F., Soria S., Pavone F. (2012). Toxic effects of amyloid fibrils on cell membranes: The importance of ganglioside GM1. FASEB J..

[161] Cecchi C., Nichino D., Zampagni M., Bernacchioni C., Evangelisti E., Pensalfini A., Liguri G., Gliozzi A., Stefani M., Relini A. (2009). A protective role for lipid raft cholesterol against amyloid-induced membrane damage in human neuroblastoma cells. Biochim. Biophys. Acta.

[162] Seghezza S., Diaspro A., Canale C., Dante S. (2014). Cholesterol drives Aβ (1–42) interaction with lipid rafts in model membranes. Langmuir.

[163] Habchi J., Chia S., Galvagnion C., Michaels T.C., Bellaiche M.M., Ruggeri F.S., Sanguanini M., Idini I., Kumita J.R., Sparr E. (2018). Cholesterol catalyses Aβ42 aggregation through a heterogeneous nucleation pathway in the presence of lipid membranes. Nat. Chem..

[164] Lorenzen N., Nielsen S.B., Yoshimura Y., Vad B.S., Andersen C.B., Betzer C., Kaspersen J.D., Christiansen G., Pedersen J.S., Jensen P.H. (2014). How epigallocatechin gallate can inhibit α-synuclein oligomer toxicity in vitro. J. Biol. Chem..

[165] Scarff C.A., Almeida B., Fraga J., Macedo-Ribeiro S., Radford S.E., Ashcroft A.E. (2015). Examination of ataxin-3 aggregation by structural mass spectrometry techniques: a rationale for expedited aggregation upon polyglutamine expansion. Mol. Cell. Proteom..

[166] Lupton C.J., Steer D.L., Wintrode P.L., Bottomley S.P., Hughes V.A., Ellisdon A.M. (2015). Enhanced molecular mobility of ordinarily structured regions drives polyglutamine disease. J. Biol. Chem..

[167] Masino L., Nicastro G., Calder L., Vendruscolo M., Pastore A. (2011). Functional interactions as a survival strategy against abnormal aggregation. FASEB J..

[168] Zhang T., Zhang J., Derreumaux P., Mu Y. (2013). Molecular mechanism of the inhibition of EGCG on the Alzheimer Aβ1–42 dimer. J. Phys. Chem. B.

[169] Tarus B., Nguyen P.H., Berthoumieu O., Faller P., Doig A.J., Derreumaux P. (2015). Molecular structure of the NQTrp inhibitor with the alzheimer Aβ1-28 monomer. Eur. J. Med. Chem..

[170] Doig A.J., Derreumaux P. (2015). Inhibition of protein aggregation and amyloid formation by small molecules. Curr. Opin. Struct. Biol..

[171] Chebaro Y., Jiang P., Zang T., Mu Y., Nguyen P.H., Mousseau N., Derreumaux P. (2012). Structures of Aβ17–42 trimers in isolation and with five small-molecule drugs using a hierarchical computational procedure. J. Phys. Chem. B.

[172] Sato M., Murakami K., Uno M., Nakagawa Y., Katayama S., Akagi K.-I., Masuda Y., Takegoshi K., Irie K. (2013). Site-specific inhibitory mechanism for amyloid-β42 aggregation by catechol-type flavonoids targeting the Lys residues. J. Biol. Chem..

[173] Ronga L., Langella E., Palladino P., Marasco D., Tizzano B., Saviano M., Pedone C., Improta R., Ruvo M. (2007). Does tetracycline bind helix 2 of prion? An integrated spectroscopical and computational study of the interaction between the antibiotic and α helix 2 human prion protein fragments. Proteins.

[174] Stoilova T., Colombo L., Forloni G., Tagliavini F., Salmona M. (2013). A new face for old antibiotics: Tetracyclines in treatment of amyloidoses. J. Med. Chem..

[175] Jiang L., Liu C., Leibly D., Landau M., Zhao M., Hughes M.P., Eisenberg D.S. (2013). Structure-based discovery of fiber-binding compounds that reduce the cytotoxicity of amyloid beta. elife.

[176] Wang Q., Liang G., Zhang M., Zhao J., Patel K., Yu X., Zhao C., Ding B., Zhang G., Zhou F. (2014). De novo design of self-assembled hexapeptides as β-amyloid (Aβ) peptide inhibitors. ACS Chem. Neurosci..

[177] Habchi J., Chia S., Limbocker R., Mannini B., Ahn M., Perni M., Hansson O., Arosio P., Kumita J.R., Challa P.K. (2017). Systematic development of small molecules to inhibit specific microscopic steps of Aβ42 aggregation in Alzheimer’s disease. Proc. Natl. Acad. Sci. USA.

[178] Habchi J., Arosio P., Perni M., Costa A.R., Yagi-Utsumi M., Joshi P., Chia S., Cohen S.I., Müller M.B., Linse S. (2016). An anticancer drug suppresses the primary nucleation reaction that initiates the production of the toxic Aβ42 aggregates linked with Alzheimer’s disease. Sci. Adv..

[179] Scherzer-Attali R., Pellarin R., Convertino M., Frydman-Marom A., Egoz-Matia N., Peled S., Levy-Sakin M., Shalev D.E., Caflisch A., Gazit E. (2010). Complete phenotypic recovery of an Alzheimer's disease model by a quinone-tryptophan hybrid aggregation inhibitor. PLoS ONE.

[180] Minicozzi V., Chiaraluce R., Consalvi V., Giordano C., Narcisi C., Punzi P., Rossi G.C., Morante S. (2014). Computational and experimental studies on β-sheet breakers targeting Aβ1–40 fibrils. J. Biol. Chem..

[181] Kokotidou C., Jonnalagadda S.V.R., Orr A.A., Seoane-Blanco M., Apostolidou C.P., van Raaij M.J., Kotzabasaki M., Chatzoudis A., Jakubowski J.M., Mossou E. (2018). A novel amyloid designable scaffold and potential inhibitor inspired by GAIIG of amyloid beta and the HIV-1 V3 loop. FEBS Lett..

[182] Frydman-Marom A., Convertino M., Pellarin R., Lampel A., Shaltiel-Karyo R., Segal D., Caflisch A., Shalev D.E., Gazit E. (2011). Structural basis for inhibiting β-amyloid oligomerization by a non-coded β-breaker-substituted endomorphin analogue. ACS Chem. Biol..

